# Peripheral blood immune profiling reveals key signatures in newly diagnosed NK/T cell lymphoma patients

**DOI:** 10.7150/thno.132582

**Published:** 2026-06-17

**Authors:** Dahui Li, Hongyuan Zou, Mingyu Wang, Na Tian, Chuanxu Liu, Wenhao Zhang, Shiyu Jiang, Ruiliang Zhu, Xiangyu Yang, Fangxia Li, Ying Gao, Wendi Wei, Ran Jia, Zihan Jiang, Yuanhua Liu, Rong Tao, Xiaozhen Liang

**Affiliations:** 1 Department of Lymphoma and Medical Oncology, Fudan University Shanghai Cancer Center, Research Center for Lymphoma, Fudan University, Shanghai 200032, China.; 2 University of Chinese Academy of Sciences, Shanghai Institute of Materia Medica, Chinese Academy of Sciences, Shanghai 200031, China.; 3 Shanghai Institute of Infectious Disease and Biosecurity, Fudan University, Shanghai 200032, China.; 4 Department of Clinical Laboratory, Children’s Hospital of Fudan University, Shanghai 201102, China.; 5 State Key Laboratory of Cardiology, Shanghai East Hospital, Tongji University School of Medicine, Shanghai 200120, China.; 6 School of Pharmacy, East China University of Science and Technology, Shanghai 200237, China.

**Keywords:** NKTCL, PBMC, EBV, single-cell RNA sequencing, PrimeFlow

## Abstract

**Rationale:**

Natural killer/T-cell lymphoma (NKTCL) is an aggressive Epstein-Barr virus (EBV)-associated non-Hodgkin lymphoma with a poor prognosis. Recent genomic and transcriptomic studies of tumor tissues have advanced our understanding of NKTCL pathogenesis, but the systemic immune profile at initial diagnosis remains incompletely elucidated.

**Methods:**

In this study, we characterized the immune landscape of peripheral blood mononuclear cells (PBMCs) from 20 newly diagnosed NKTCL patients and 12 healthy donors using single-cell RNA sequencing and confirmed our results through flow cytometry and PrimeFlow.

**Results:**

We identified a distinct proliferative-NK/T (Proli-NK/T) cell subset in NKTCL, characterized by high expression of cell cycle-related genes but lacking a malignant phenotype. Additionally, we observed a reduction in total and classical memory B cells, accompanied by enrichment of apoptosis and cell differentiation signatures. NK cells showed increased expression of HLA class II and activation markers, along with enhanced predicted interactions with CD4^+^ T cells. The decrease of naive CD4^+^ T cells might imply their skewed differentiation into Th1 and Th17 cells, while expansion of granzyme K (GZMK^+^)-expressing CD8^+^ central memory T cells was associated with interferon-γ-driven responses. Patients with a high intracellular EBV load exhibited accumulation of highly cytotoxic CD56^dim^*_PTPRCAP* NK cells and GZMK^+^ GZMB^+^ CD8^+^ effector memory T cells, along with a marked depletion of memory B cells. This implies a correlation between intracellular EBV burden and peripheral immune dysregulation in NKTCL.

**Conclusions:**

We have uncovered the dynamic changes in PBMCs of newly diagnosed NK/T cell lymphoma patients and identified specific characteristics in patients with high intracellular EBV levels. Our findings provide new insights into the immunopathogenesis of NKTCL, offering valuable information for immune-based stratification and the development of therapeutic strategies.

## Introduction

Natural killer/T-cell lymphoma (NKTCL) is a rare, highly aggressive Epstein-Barr virus (EBV)-associated non-Hodgkin’s lymphoma that predominantly involves the upper aerodigestive tract and is more prevalent in East Asia and Latin America. It is characterized by the malignant proliferation of EBV-positive NK or cytotoxic T cells and is associated with poor clinical outcomes 1-5. Neoplastic NKTCL cells express CD2, CD56, cytoplasmic CD3, EBV-encoded small RNAs (EBERs), and cytotoxic molecules such as granzyme and perforin. The striking geographic distribution and near-universal association with EBV suggest that host genetic susceptibility and region-specific EBV strains may contribute to NKTCL pathogenesis 6, 7. EBV strain type A is the most prevalent strain in NKTCL and displays a type II latency program, which usually expresses EBERs, EBV nuclear antigen 1 (EBNA1), and latent membrane proteins (LMPs). The precise mechanism of EBV infection in NKTCL is not fully understood, but a high viral load is associated with worse outcomes and serves as an adverse prognostic factor for overall survival in NKTCL 8-11.

Clinically, NKTCL is notorious for its resistance to anthracycline-based chemotherapy, mainly due to high expression of *MDR1*-encoded P-glycoprotein 12, 13. L-asparaginase-based regimens have shown improved response rates and overall survival in early-stage patients, but long-term outcomes remain poor in advanced-stage patients. Immune therapy has become a transformative and promising strategy for treating NKTCL. Blockade with the PD-1/PD-L1 checkpoint yields encouraging results in some relapsed/refractory (R/R) patients 14-17. Autologous EBV-specific T cells have also shown efficacy in advanced NKTCL patients 3, 18. Despite the significant advancements in NKTCL treatment over the past decade, many patients still experience relapse or refractory disease, and durable remissions remain uncommon, especially in advanced-stage or relapsed patients. This underscores the critical need for deeper insights into NKTCL lymphomagenesis and host-tumor immune interactions.

Recent multi-omics studies of NKTCL tumor biopsies have substantially advanced our understanding of its molecular pathogenesis. Comprehensive genetic profiling has identified recurrent alterations and mutations in pathways such as JAK-STAT, MAP kinase signaling, tumor suppressor genes, and epigenetic regulators, as well as mutations affecting immune surveillance, thereby providing essential clues for therapeutic targeting 19-27. Parallel analyses of EBV genomes in NKTCL have revealed diverse structural variations and single-nucleotide variants, including recurrent changes in lytic genes such as* BALF3*, together with high *BALF3* expression in tumor transcriptomes 20, 28. These findings highlight the complex interplay between tumor intrinsic factors (e.g., genetic mutations and deficiencies), viral factors, and the human immune system in NKTCL. However, almost all such studies have focused on tumor tissues, and the systemic immune landscape, particularly at initial diagnosis, remains poorly characterized.

Emerging evidence across multiple cancer types suggests that immunoprofiling of peripheral blood mononuclear cells (PBMCs) can predict treatment response and guide biomarker-driven clinical trials 29-33. In NKTCL, systematic characterization of PBMC immune composition and functional states is lacking. In this study, we utilized single-cell RNA sequencing (scRNA-seq), PrimeFlow, and multiparameter flow cytometry to comprehensively delineate the phenotypic and transcriptional landscape of PBMCs from newly diagnosed NKTCL patients. Our work defines a peripheral blood immune profile of NKTCL at initial diagnosis and provides a framework for future studies of EBV-driven immune dysregulation and for refining immunotherapeutic strategies in this disease.

## Methods

### Sample selection and preparation

For this study, peripheral blood was obtained from 12 healthy donors and 20 patients newly diagnosed with NKTCL at the Fudan University Cancer Center. The study was approved by the Cancer Center (050432-4-1911D) and Children’s Hospital Review Board of Fudan University (2022-1). Informed consent was obtained in accordance with the Declaration of Helsinki. Additional information, including the age and sex of participants, is included in supplemental Table S1.

### Blood sample processing and PBMC isolation

Fresh peripheral blood samples were collected into EDTA-coated tubes from patients or healthy donors and processed within 2 h of collection. After centrifugation at 1800 rpm for 5 min at room temperature (ramp-up 6, ramp-down 0), the plasma supernatant was harvested and stored at -80 ℃. The remaining part was diluted with 1× PBS at 1:1 ratio, overlaid on Ficoll-Paque (Cytiva, 17144002), and centrifuged at 1800 rpm for 20 min at room temperature (ramp-up 6, ramp-down 0). PBMCs were collected from PBS - Ficoll-Paque interlayer and lysed red blood cells with red blood cell lysis buffer for 5 min at room temperature, then washed twice in 1× PBS. The freshly prepared cells were then used in subsequent assays for further analysis.

### Single-cell RNA sequencing

The freshly collected PBMCs for scRNA-seq were resuspended in 1× PBS supplemented with 0.04% BSA at a final concentration of 1000 cells/μL. After passing the quality control inspection, the cell suspension was loaded into a 10× Chromium Chip (v3.1 PN:1000120) and barcoded using a 10× Chromium Controller. RNA from the barcoded cells was then reverse-transcribed, amplified, and prepared into sequencing libraries with the 10× Library Construction Kit (v3.1 PN:1000190) according to the manufacturer’s instructions. Sequencing was carried out on an Illumina NovaSeq with 150-bp paired-end reads at Novogene Bioinformatics Technology Co., Ltd (Shanghai, China).

### Data processing, correction, and integration

Raw scRNA-seq data were initially pre-processed using CellRanger (version 8.0.1, 10× Genomics) to align reads to the human genome (GRCh38, 2024-A from 10× Genomics) and count the unique molecular identifiers (UMIs) for each gene to generate specific gene cell count tables. For each scRNA-seq sample, the count tables were filtered to retain the genes detected in at least 10 cells and cells with a minimum gene count of 300. Doublets were removed using the R package scDblFinder 34, which generates artificial doublets between clusters. Clusters were identified using functions embedded in the R package Seurat v5 as follows: 1) cells were filtered based on total RNA count (nCount_RNA < 60000), total number of genes (300 < nFeature_RNA < 6000), and mitochondrial percentage (percent.MT < 20); 2) gene counts were log-normalized by scaling to 5000 counts (scale factor = 5000) and log-transformed for each cell; 3) k-nearest neighbors (20) were calculated for each cell and a graph was generated for all cells; 4) the original Louvain algorithm was used for modularity optimization on the graph to identify cell modules.

The cleaned data from 32 samples, after doublet removal, were combined, and variable genes across all samples were selected using "vst" for PCA dimension reduction (60 pcs). The PCA embeddings were corrected using harmony to account for individual effects. Based on the corrected PC embeddings, k-means nearest neighbors and Louvain modularity optimization were sequentially performed to identify clusters. To visualize the cell clusters, the first 30 corrected PCs were used for further dimension reduction with Uniform Manifold Approximation and Projection.

### Cluster annotation

Cluster annotation was conducted for the main cell type in PBMC using ScType 35. Cluster-specific differentially expressed marker genes were selected and listed in supplemental Table S2, primarily based on comprehensive published literature searches targeting key markers of interest 36, 37 and differential expression analysis.

### Trajectory analysis

Single cell trajectories of CD8^+^ T cells, CD4^+^ T cells, and NK cells were analysed using the Monocle3 38. We converted the Seurat data objects into CellDataSet objects required by the Monocle3 framework. This allowed for cluster assignment and reduced dimension “umap” space for trajectory learning. Principal graphs were built via reversed graph embedding without any partitioning. Manual assignment was performed to set the trajectory root using the subtype representing the initial phase of each cell type.

### Pathway and gene set enrichment analysis

Pseudobulk data were calculated for each subtype of major cell subsets from individual PBMC samples. Differential expression genes (DEGs) enrichment was analyzed using DESeq2. The output “stat” ranked genes were further input to GSEA (4.3.3, https://www.gsea-msigdb.org/) for KEGG (human Legacy genesets, v2024.1) enrichment analysis. GO enrichment analysis was carried out by using R software (v.4.2.2) package clusterProfiler (v.4.5.0) 39 through Hiplot Pro (https://hiplot.com.cn/), a comprehensive web service for biomedical data analysis and visualization.

### Cell-to-cell communication

The crosstalk between NK and CD4^+^ T cells was estimated using CellChat 2 38, where any two subtypes with more than 10 cells were involved to compute the probability of interaction. The interactions were determined as significant if the *p*-value was less than 0.05. The probability values from all NKTCL and healthy donors were further analyzed to extract the crosstalk feature in diseases.

### Genomic instability analysis

The number of NK and T cell CNVs was analyzed using the R package inferCNV (https://github.com/broadinstitute/inferCNV), randomly selecting NK/T cells in our healthy controls as a reference. For a reasonable inference, the cell numbers of the subtypes were balanced. Essentially, we downsampled the cells in each subtype to the minimum number involved if the minimum number is less than 1000, to 1000 otherwise.

### Heatmap and volcano plots

The heatmaps were produced with R software (v.4.2.2) package “pheatmap” (v.1.0.12) 40 and volcano plots were generated using R software (v.4.2.2) package “ggpubr” (v0.4.0) 41 and “ggplot2” (v3.4.2) 42 through Hiplot Pro (https://hiplot.com.cn/).

### PrimeFlow assays

EBV specific RNA probes targeting* EBER1/2*-Alexa Fluor 647 (Invitrogen, PF-210, VF1-12409) and *gp350*-Alexa Fluor 570 (Invitrogen, PF-210, VP47VWR) were used to detect EBV-infected cells and EBV-reactivating cells, respectively. Three million PBMCs per sample were stained with specific antibodies for CD3-FITC (Biolegend, 317305), CD8-PB (Biolegend, 344717), CD56-BV650 (Biolegend,318344), and CD19-PE-Cy7 (eBioscience, 25-0199-42), followed by labeling and staining with targeted RNA probes for *EBER1/2* and *gp350* according to the manufacturer’s instructions (PrimeFlow™ RNA Assay Kit, Invitrogen, 88-18005-210). Cells were acquired using the Celesta flow cytometer (BD Biosciences) and analyzed with FlowJo software.

### Flow cytometry and intracellular staining

Isolated PBMCs were resuspended and washed with FACS buffer (1× PBS supplemented with 2% FBS), and then blocked with blocking buffer (CD16/CD32 antibody in FACS buffer at a 1:200 dilution, BD Biosciences, 564220) for 30 min at 4 ℃. After blocking, the cells were washed with FACS buffer three times and stained with flow cytometry antibodies for 30 min at 4 ℃. Subsequently, the cells were washed three times and fixed with 4% paraformaldehyde (PFA) buffer. The cells were then acquired on a Celesta flow cytometer (BD Biosciences) and analyzed using FlowJo software.

For intracellular staining, cells were first stained with surface markers and then fixed with 4% PFA for 15 min at 4 ℃. After fixation, the cells were permeabilized with BD fixation/permeabilization solution (BD Pharmingen, 554715) and washed once with 1× washing buffer (10× washing buffer diluted by ddH_2_O). The cells were subsequently incubated with intracellular staining antibodies on ice in the dark for 1h. Following three washes, the cells were obtained on the Celesta flow cytometer (BD Biosciences), and the data analysis were conducted using FlowJo software.

CD19-PE-Cy7 (eBioscience, 25-0199-42), CD27-PE (Biolegend, 356406), CD38-APC (Biolegend, 356606), IgD-APC-Cy7 (Biolegend, 348218), CD10-BV421 (Biolegend, 312218), CD95(FAS)-FITC (Biolegend, 305605), Cleaved Caspase3-PB (Cell Signaling Technology, 8788S), and PRDM1/BLIMP1-AF488 (Novus, NB600-235 AF488) were used for B cell subset staining; CD3-PE (Biolegend, 300408), CD56-BV650 (Biolegend, 318344), HLA-DR,DP,DQ-FITC (Biolegend, 361705), CD11a/CD18 (LFA-1)-FITC (Biolegend, 363416), CD335(NKp46)-PB (Biolegend, 331912), CD336(NKp44)-PE (Biolegend, 325107), CD337(NKp30)-APC (Biolegend, 325209) were used for NK cell subset staining.

### NK cell isolation and cytotoxicity assay

NK cells were negatively isolated from PBMCs of healthy donors and NKTCL patients using the human NK Cell Isolation Kit (Miltenyi Biotec, 130-092-657) following the manufacturer’s instructions. K562 target cells were labeled with CFSE. Specifically, K562 cells were resuspended in 1× PBS supplemented with 0.1% FBS at 2×10^6^ cells/mL and a final concentration of 1 mM CFSE. The cells were then incubated at 37 ℃ for 15 min, followed by the addition of the same volume of pre-warmed FBS and another 15 min incubation at 37 ℃. Finally, the cells were washed three times with 1× PBS supplemented with 2% FBS and resuspended in complete medium. The labeled K562 cells were co-cultured with sorted NK cells at an effector:target cell ratio of 5:1 for 4 h.

Two approaches were used to assess NK cell cytotoxicity. The first approach measured cytotoxic activity of NK cells through CD107a expression: at the start of co-culture (0 h), anti-human CD107a-PE-Cy7 antibody (BD Biosciences, 561348) was added to the culture medium (1:200), and Golgi stop was added after 1 h. CD107a expression levels were analyzed by flow cytometry, and CD56-BV605 (Biolegend, 318334) was used for NK cell gating. The second approach assessed the cytotoxic activity of NK cells by determining the CFSE^+^ K562 cell death rate: after 4 h of co-culture, cells were stained with the LIVE/DEAD™ Fixable Aqua Dead Cell Stain Kit (Invitrogen, L34966) and analyzed by flow cytometry. As controls, K562 cells were incubated either in medium without NK cells (spontaneous death) or with 0.1% Tween (maximum death).

### CD8^+^ T cell activity assay

Total PBMCs were cultured in RPMI 1640 medium with L-glutamine (Gibco, 11875093) supplemented with 10% FBS and 1% penicillin/streptomycin at a concentration of 2×10^6^ cells/mL in a 24-well plate. The PBMCs were stimulated for 24 h by using human anti-CD3/CD28 DynaBeads following the manufacturer’s instructions (Gibco, 11161D), and Golgi stop was added during the last 6 h of stimulation. Stimulated PBMCs were then collected and analyzed by flow cytometry. CD3-APC-Cy7 (Biolegend, 300426), CD56-BV605 (Biolegend, 318334), CD4-PE (Biolegend, 300507), CD8-BV421 (Biolegend, 344747), Granzyme B-APC (Biolegend, 372203), and IFN-γ-PE-Cy7 (Biolegend, 502527) were used for CD8^+^ T cell gating and cytotoxicity analysis.

More information on key resources was shown in supplemental Table S3.

### Statistical analysis

Statistical analysis was conducted using the stats package in R for scRNA-seq data and GraphPad Prism for flow cytometry. A linear model was utilized, incorporating multiple factors when necessary, along with unpaired two-tailed Student’s t-tests or Wilcoxon tests as appropriate. Compositional analysis of cell-type fractions was performed using scCODA, with FDR-adjusted *p*-values for untargeted analyses such as differentially expressed genes or pathways. In the flow cytometry assay for PBMC composition validation, the *p* values were determined by a paired two-tailed Student’s t-test. A *p* value of < 0.05 was considered statistically significant. Specific or additional information on the statistical tests was provided in the figure captions corresponding to each figure.

## Results

### Study design and immune cell type definition

**Figure 1:**
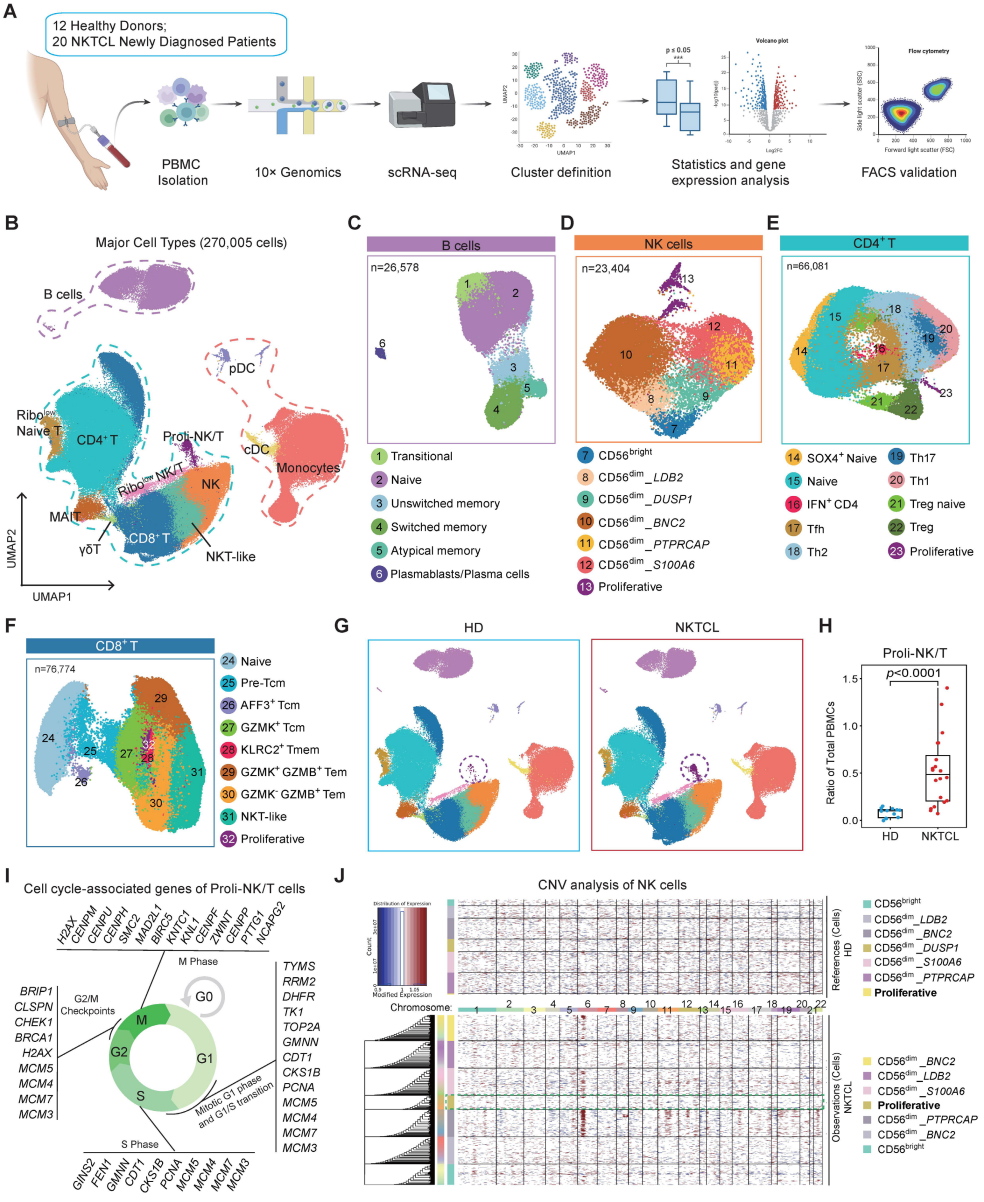
** Single cell profiling of PBMCs in newly diagnosed NKTCL patients.** (A) Experimental design overview of the study. Created in BioRender. Zou, H. (2026) https://BioRender.com/ruiwe3i. (B) UMAP plot showing the major cell types identified within PBMCs from 32 samples. (C-F) UMAP plots of B, NK, CD4^+^ T, and CD8^+^ T cell subsets identified by clustering. (G) UMAP plots displaying changes in major cell types between HD and NKTCL groups. (H) Boxplots of Proli-NK/T cell ratio in total PBMCs. Hinges represent the 25th and 75th percentiles, and whiskers extend to values within 1.5 times the interquartile range (1.5× IQR) from the hinges. Horizontal bars indicate the median value. The *p* value was determined by an unpaired two-tailed Student’s t-test, *p* < 0.05 was considered statistically significant. (I) Cell cycle-associated genes of Proli-NK/T cells presented in cycling phases. (J) Heatmap of CNV analysis of NK cell subsets. Red and blue colors represent high and low CNV level, respectively.

To investigate the changes in PBMC composition between newly diagnosed NKTCL patients and healthy donors, we collected peripheral blood samples from 12 healthy donors and 20 NKTCL patients. We isolated PBMCs for single-cell sequencing analysis, resulting in a total of 270,005 cells after filtering out low-quality droplets (Figure 1A; Table S1). We identified 13 major cell types belonging to three subpopulations (Figure 1B, S1A, and S1B; Table S2): B cell population (purple line, expressing* CD79A, IGHD, IGHG1, etc.*), myeloid cell population (orange line) including monocytes (expressing* CD14, FCGR3A, CD68, etc.*), conventional dendritic cells (cDC, expressing* CD1C, CLEC10A, etc.*), and plasmacytoid dendritic cells (pDC, expressing* IL3RA, CLEC4C, etc.*), and NK&T cell population (blue-green line) including CD4^+^ T (expressing* CD3D, CD3E, CD4, etc.*), CD8^+^ T (expressing* CD3D, CD3E, CD8A, CD8B, etc.*), natural killer (NK, expressing* NCAM1, NKG7, etc.*), NKT-like (expressing* CD3D, CD3E, NCAM1, KLRC2, etc.*), mucosal-associated invariant T (MAIT, expressing *CD3D, CD3E, SLC4A10, etc.*), γδT (expressing *CD3D, CD3E, TRDV2, TRDV9, etc.*), proliferative-NK/T (Proli-NK/T, expressing *MKI67, TYMS* with *CD4* or *CD8A* or *NCAM1*). Additionally, two subsets expressing lower levels of ribosome-associated genes (*RPS7 etc.*) were identified in the NK&T cell population: Ribo^low^ Naive T (expressing *CD3, CD4, CD8,* and naive T cell markers: *CCR7, TCF7, etc.*), and Ribo^low^ NK/T (expressing* CD3, CD4, CD8, NCAM1,* and effector cell markers: *PRF1, GZMB, etc.*).

Subsequently, B, NK, CD4^+^ T, and CD8^+^ T (including NKT-like) cells were further subclustered into 32 distinct subsets, and Proli-NK/T cells were divided into NK, CD4^+^ T, and CD8^+^ T cell subsets based on *NCAM1*, *CD4*, or *CD8* marker gene expression, named "Proliferative" subsets. Among 26,578 B cells, we identified 6 major clusters, including transitional B cells (expressing *CD38, IGHD, IGHM, MME* (*CD10*), *etc.* without* CD27*), which are early-stage cells exiting from bone marrow, naive cells (expressing *CD38, IGHD, ZBTB16, etc.* without* CD27*), unswitched memory B cells (expressing *CD27*, *IGHD, IGHM, etc.* without* CD38*), switched memory B cells (expressing *CD27*, *SSPN, etc.* without* CD38* and* IGHD*), atypical memory B cells (expressing *CD1C, SOX5, FCRL5, FCRL3, etc.*), and plasmablasts/plasma cells (expressing high *CD27,* high* CD38, PRDM1, XBP1, etc.*) (Figure 1C, and S1C; Table S2). We divided the NK cell population into 3 major subpopulations: CD56^bright^ (higher expression level of *NCAM1*), CD56^dim^ (high expression level of *FCGR3A*), and proliferative (*TYMS*) NK cells, and CD56^dim^ NK cells were also further classified into 5 subsets based on their specific markers (Figure 1D, and S1D; Table S2).

CD4^+^ T cells comprised 66,081 cells and were divided into 10 distinct subsets (Figure 1E, and S1E; Table S2): naive cells (*CCR7*, *LEF1 etc.*), SOX4^+^ naive cells (expressing *SOX4* with naive cell markers), Tfh (*CXCR5, PDCD1 etc.*), Th1 (*GZMK*, *CCL5*,* CXCR3 etc.*), Th17 (*CCR6*, *RORC*, *AHR etc.*), Th2 (*CCR4*, *GATA3 etc.*), Treg (*FOXP3*, *IL2RA, IKZF2 etc.*), Treg naive (Treg bulk signature with naive cell markers *LEF1*, *CCR7, IGF1R etc.*), IFN^+^ CD4 (*IFIT1*, *IFIT3 etc.*), and proliferative (*MKI67, TYMS etc.*) CD4 T cells. For the CD8^+^ T and NKT-like cell cluster refining, we totally revealed 9 subpopulations (Figure 1F, and S1F; Table S2). CD8^+^ naive T cells expressed naive cell markers (*CCR7*, *SELL*, *LEF1*, and *TCF7* at higher levels than central memory T (Tcm) cells, and without *FAS* expression). Tcm cells included 3 subsets: GZMK^+^ Tcm (*GZMK*, *CD27*, *CD28 etc.*), AFF3^+^ Tcm (*AFF3*, *HAVCR2*, *GPR183 etc.*), and pre-Tcm in which the expression levels of *LEF1* and *TCF7* were between GZMK^+^ Tcm and naive cells. Compared to Tcm population,* GNLY*, *PRF1* and *NKG7* were more abundantly expressed in effector memory T (Tem) cells, including GZMK^+^ GZMB^+^ Tem, GZMK^-^ GZMB^+^ Tem, and KLRC2^+^ T memory (Tmem) cells. NKT-like cells expressed *NCAM1* and *ZBTB16*, and proliferative subset was defined by *MKI67* and* TYMS*.

### Immune profiling of newly diagnosed NKTCL patients

Comparison of PBMC composition between NKTCL and healthy donor (HD) groups revealed a significant accumulation of Proli-NK/T cells in NKTCL patients (Figure 1G and 1H). Enrichment analysis with the STRING database displayed the top 50 expressed genes (avg_log2FC >1 and pct.2 < 0.3) of Proli-NK/T cells (Figure S2A), most of which were associated with cell cycle processes including G1 phase, G1/S transition, S phase, G2/M, and M phase (Figure 1I, S2A, and S2B). To further examine whether these highly proliferative NK and T cells were malignant tumor cells, we analyzed CNV scores in NK, CD4^+^ T, and CD8^+^ T cell subsets. No significant difference in CNV scores was observed for proliferative-NK, CD4^+^ T, and CD8^+^ T cells between NKTCL patients and healthy donors (Figure 1J and S2C), indicating that these highly proliferative cells were normal immune cells and not malignant proliferating tumor cells. Additionally, Proli-NK/T cells downregulated the N-Glycan biosynthesis signaling pathway and upregulated genes in response to interferon-gamma (IFN-γ) stimulation (Figure S2D and S2E). We hypothesize that Proli-NK/T cells could be a naturally occurring population of rapidly dividing cells stimulated by the abundant presence of IFN-γ in the peripheral microenvironment of NKTCL patients.

### Classical memory B cell reduction in NKTCL PBMCs is associated with cell death and differentiation

**Figure 2:**
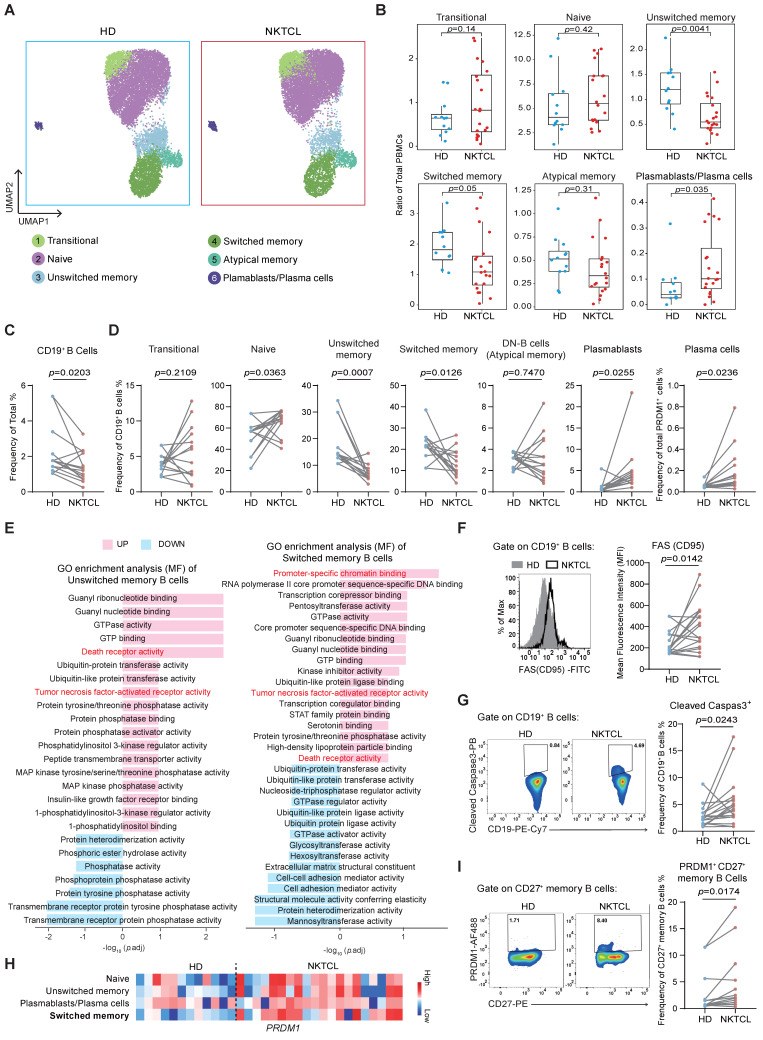
** Memory B cell reduction linked to cell death and differentiation in NKTCL PBMCs.** (A) UMAP plots displaying changes in B cell subsets between HD and NKTCL groups. (B) Boxplots illustrating the ratio of each B cell subset in total PBMCs in HD and NKTCL groups. (C) FACS analysis showing the frequency of CD19^+^ B cells in total PBMCs in HD (n=7) and NKTCL (n=14) groups. (D) Flow cytometry analysis showing the frequency of B cell subsets from CD19^+^ B cells or total PRDM1^+^ cells in HD (n=7) and NKTCL (n=14) groups. Transitional (CD27^-^ IgD^+^ CD10^+^), naive B cells (CD27^-^ IgD^+^ CD10^-^), unswitched memory (CD27^+^ IgD^+^), switched memory (CD27^+^ IgD^-^), double-negative (DN) cells (or atypical memory B cells, CD27^+^ IgD^-^), plasmablasts (CD19^+^ CD27^high^ CD38^high^), and plasma cells (PRDM1^+^ CD27^high^ CD38^high^). (E) Bar plots displaying GO enrichment analysis of molecular function (MF) signaling pathways for comparing the changes in NKTCL vs. HD in unswitched memory and switched memory B cell clusters. (F) Flow cytometry analysis of FAS (CD95) expression in CD19^+^ B cells in HD (n=11) and NKTCL (n=18) groups. (G) Flow cytometry analysis of percentage of Cleaved Caspase3^+^ cells in CD19^+^ B cells in HD (n=11) and NKTCL (n=18) groups. (H) Heatmap showing *PRDM1* expression in naive, unswitched memory, plasmablasts/plasma cells, and switched memory B cells. (I) Flow cytometry analysis showing the frequency of PRDM1^+^ CD27^+^ memory B cells in HD (n=7) and NKTCL (n=14) groups. In (B), hinges represent the 25th and 75th percentiles, and whiskers extend to values within 1.5× IQR from the hinges. Horizontal bars indicate the median value. The *p* values were determined by unpaired two-tailed Student’s t-test. In (C), (D), (F), (G), and (I), the *p* values were determined by paired two-tailed Student’s t-test. *p* < 0.05 was considered statistically significant.

In the B cell subset, there was a significant decrease in both unswitched memory and switched memory B cells (classical memory B cells) in total PBMCs, along with an increased ratio of plasmablasts/plasma cells (Figure 2A and 2B). The ratio of naive B cells also increased in total B cells (quantity standardized) due to the decrease of classical memory B cells (Figure S3A). Flow cytometry analysis revealed the reduction of CD19^+^ B cells in total PBMCs and further confirmed the decreased ratio of unswitched memory (CD27^+^ IgD^+^) and switched memory (CD27^+^ IgD^-^) B cells, as well as the increased ratio of naive B cells (CD27^-^ IgD^+^ CD10^-^), plasmablasts (CD19^+^ CD27^high^ CD38^high^) in total CD19^+^ B cells and plasma cells (PRDM1^+^ CD27^high^ CD38^high^) in total PRDM1^+^ cells (Figure 2C, 2D, and S3B).

To further investigate the mechanism of composition alteration in the B cell subset, we compared the differentially expressed genes between NKTCL and HD groups (Figure S3C). Enrichment analysis showed upregulation of "Death receptor activity" and "Tumor necrosis factor-activated receptor activity" signaling pathways in naive, unswitched memory, and switched memory B cells, with increased *FAS (CD95)* expression (Figure 2E and S3D). Flow cytometry further confirmed higher FAS expression (Figure 2F and S3B) and more cleaved Caspase3 in CD19^+^ B cells (Figure 2G and S3B) in NKTCL patients, indicating that these cells were prone to FAS-mediated apoptosis, which might contribute to the reduction of B cells in NKTCL patients.

Additionally, the expression of *PRDM1* and the enriched pathway of "Promoter-specific chromatin binding" were also upregulated in switched memory B cells (Figure 2E and 2H), indicating that these memory B cells tended to differentiate into plasmablasts/plasma cells. Flow cytometry further confirmed the accumulation of PRDM1^+^ CD27^+^ classical memory B cells (Figure 2I and S3B), consistent with the increase of plasmablasts and plasma cells in NKTCL patients.

### Increased HLA class II expression and CD4^+^ T cell communication are distinctive features of NK cells in NKTCL PBMCs

We further investigated the characteristics of NK and T cell populations in NKTCL patients. Analysis of the CD8^+^ T cell subset revealed a significant increase in the ratio of GZMK^+^ Tcm cells within total CD8^+^ T cells (Figure S4A-S4C). Differential gene expression and GO enrichment analysis comparing NKTCL and HD groups showed that GZMK^+^ Tcm cells primarily responded to IFN-γ and played a role in antiviral defense by expressing *PARP9*, *STAT1*, *GBP1*, and *IFI27* (Figure S4D and S4E). These signaling pathways were also enriched in GZMK^-^ GZMB^+^ Tem and NKT-like cells (Figure S4F and S4G).

**Figure 3:**
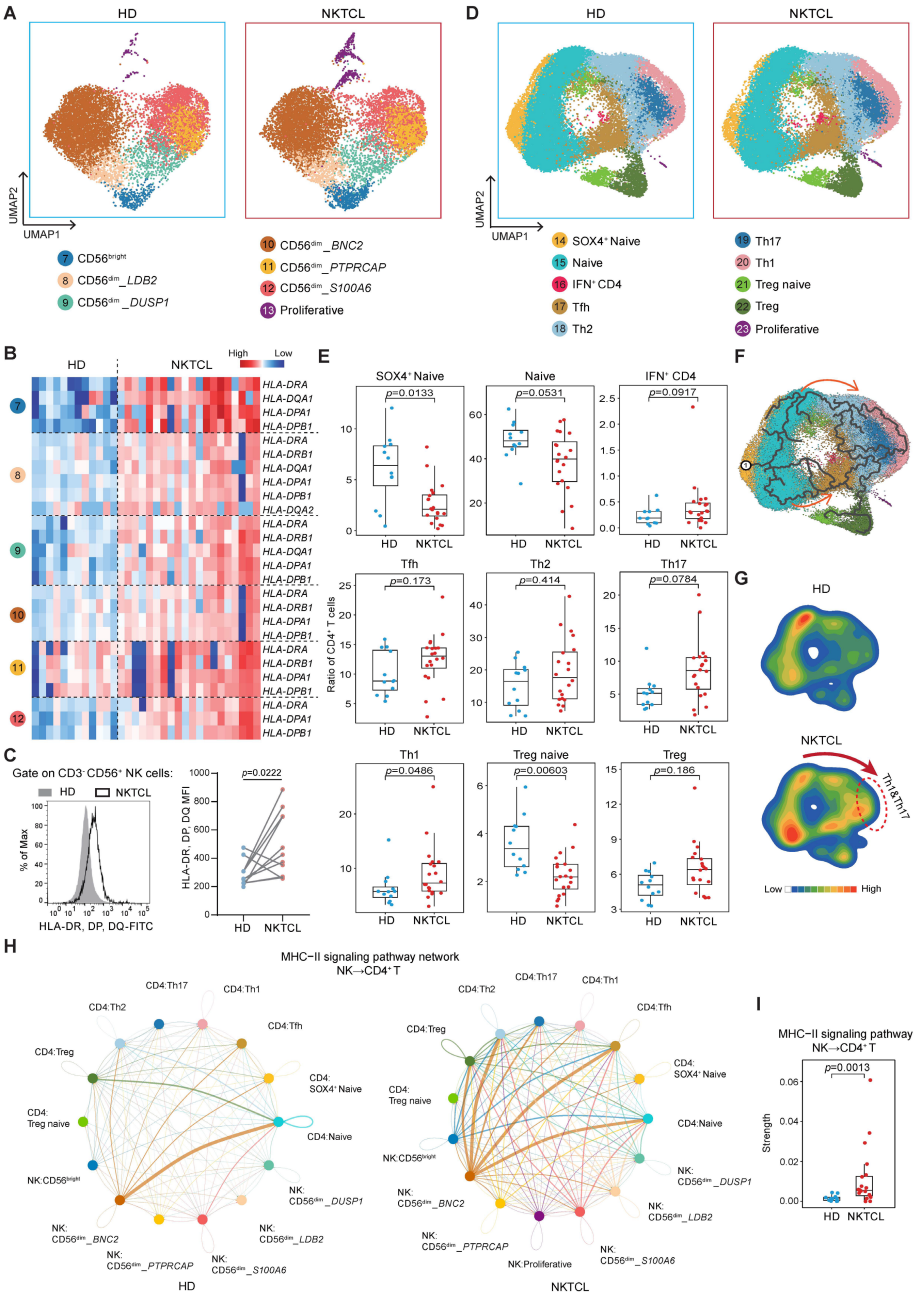
** Enhanced HLA Class II expression and CD4^+^ T cell communication as NK cell signatures in NKTCL PBMCs.** (A) UMAP plots showing changes in NK cell subsets between HD and NKTCL groups. (B) Heatmap displaying the expression of HLA Class II-associated genes in NK cell subsets. (C) FACS analysis showing the expression of HLA-DR, DP, and DQ in NK cells in HD (n=7) and NKTCL (n=11) groups. (D) UMAP plots illustrating changes in CD4^+^ T cell subsets in HD and NKTCL groups. (E) Boxplots presenting the ratio of each subset in CD4^+^ T cells in HD and NKTCL groups. (F) UMAP plot depicting the developmental trajectory of CD4^+^ T cells. (G) UMAP density plots characterizing the distribution of CD4^+^ T cells in HD and NKTCL groups. (H) Circle plots showing cell-cell contact between NK and CD4^+^ T cells through the MHC-II signaling pathway in HD and NKTCL groups. (I) Boxplots showing the strength statistic of NK-CD4^+^ T cell communication through the MHC-II signaling pathway in HD and NKTCL groups. In (C), the *p* value was determined by paired two-tailed Student’s t-test. In (E) and (I), hinges represent the 25th and 75th percentiles, and whiskers extend to values within 1.5× IQR from the hinges. Horizontal bars indicate the median value. In (E), the *p* values were determined using a general linear model with the effect of age involved; in (I), the *p* values were determined by unpaired two-tailed Student’s t-test. *p* < 0.05 was considered statistically significant.

The ratio of NK cell subsets had no significant difference between NKTCL and HD groups, except for an increased ratio of CD56^bright^ cells in total NK cells (Figure 3A, S5A, and S5B). However, a common feature was observed in all NK cell subsets: as exemplified in CD56^bright^ cells, HLA Class II genes and the "MHC class II" signaling pathway were upregulated in NKTCL patients (Figure S5C and S5D), and the heatmap displayed significant variation of HLA Class II gene expression in other clusters between NKTCL and HD groups (Figure 3B). Flow cytometry analysis further revealed that the NK cell frequency in lymphoid cells did not change, but there was an increased expression of activation markers NKp46 and NKp30 (Figure S5E, S5F, and S5G), as well as HLA class II molecules HLA-DR, DP, and DQ (Figure 3C and S5E). The upregulation of HLA Class II genes could be due to IFN-γ-driven effects, as accompanied by the upregulation of other IFN-γ responsive genes *GBP5* and *STAT1* in NK cell subsets (Figure S5H). Li et al. noticed that some patients had a high transcription level of HLA Class II genes in malignant NK cells with EBV infection of NKTCL tumors and had a high degree of CNVs at the MHC region encompassing HLA-II genes in chromosome 6 43. Our analysis did not reveal the presence of malignant proliferating tumor cells in PBMCs, but we did observe that CNVs score was slightly increased in chromosome 6 of NK cells in PBMCs of NKTCL as compared to PBMCs of HD groups (Figure 1J). This distinction might result from the interplay between EBV infection and genetic predisposition.

Furthermore, we found a significant decrease in the ratio of SOX4^+^ naive and naive CD4^+^ T cells in total CD4^+^ T cells (Figure 3D, 3E and S6A), with an upregulated enrichment of the "T cell differentiation" associated signaling pathway (Figure S6B). Th1 cells significantly accumulated, and Th17 cells slightly increased in NKTCL patients, while the ratio of Tfh and Th2 cells in total CD4^+^ T cells showed no difference between NKTCL and HD groups (Figure 3E). Combined with the developmental trajectory, we reasoned that naive CD4^+^ T cells in NKTCL patients might mainly differentiate into Th1 and Th17 cells (Figure 3F and 3G), responding to interferon-gamma and involved in interleukin-2 production (Figure S6C).

Cells expressing HLA Class II molecules are known to interact with CD4^+^ T cells, we next analyzed cell-to-cell communication between NK and CD4^+^ T cells. NK cells with upregulated HLA Class II gene expression showed an enhanced communication level with CD4^+^ T cells through the MHC-II signaling pathway in NKTCL patients compared to the HD group (Figure 3H and 3I).

### Enhanced immune dysregulation in PBMCs of NKTCL with high levels of intracellular EBV

**Figure 4:**
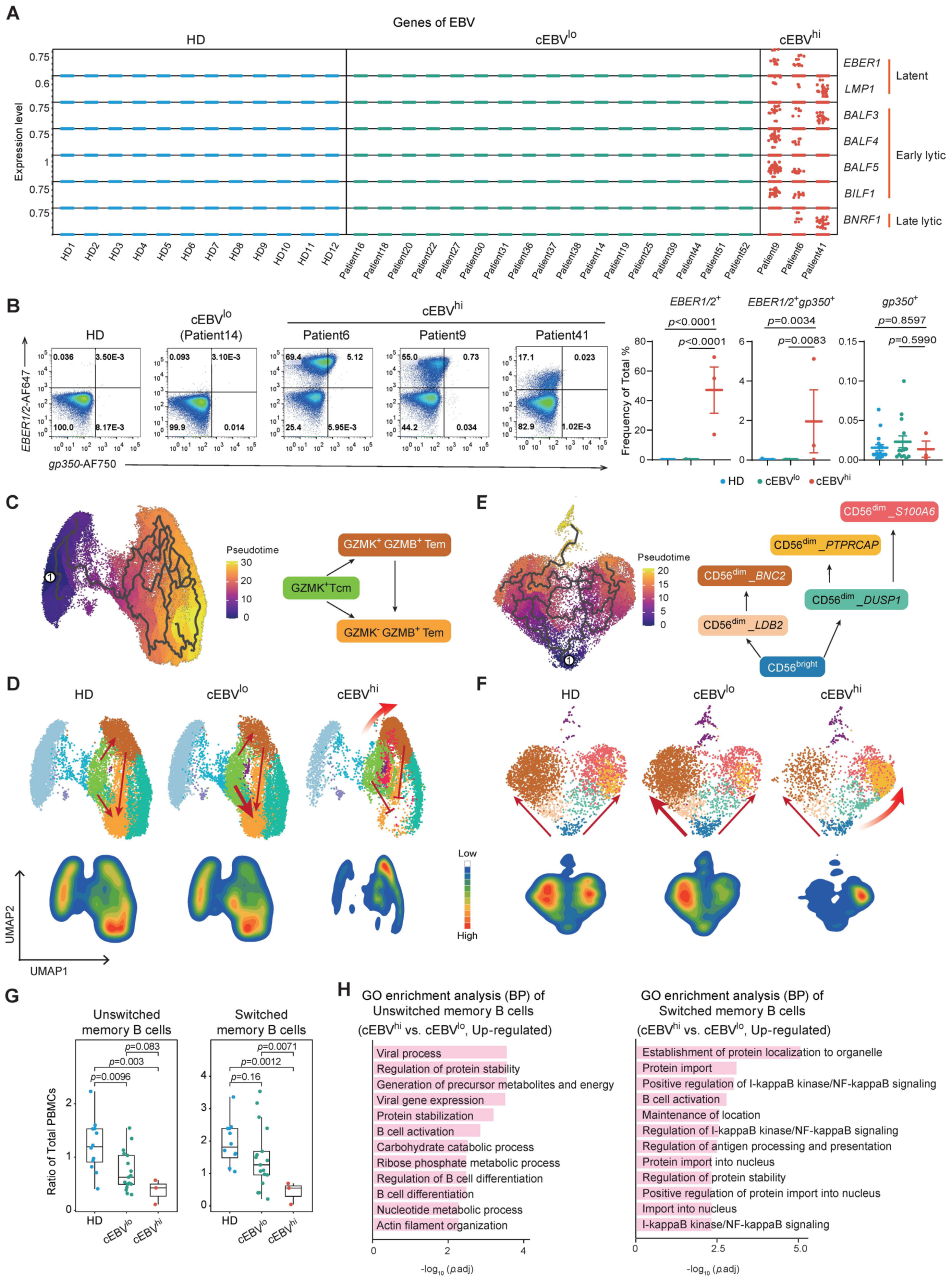
** Enhanced immune dysregulation linked to high levels of intracellular EBV in NKTCL PBMCs.** (A) Detection of EBV gene expression in PBMCs using scRNA-seq. The reference sequence of EBV genome used for EBV transcript detection in scRNA-seq analysis was derived from NCBI: Human herpesvirus 4 complete wild type genome, GenBank: AJ507799.2. (B) PrimeFlow assay showing the expression of *EBER1/2* or/and *gp350* from total PBMCs in HD (n=17), cEBV^lo^ (n=14, matching with the samples in scRNA-seq analysis but without patient 31, 39 and 44), and cEBV^hi^ (n=3, matching with the samples in scRNA-seq analysis) groups. Data were presented as mean ± s.e.m; the *p* values were determined by unpaired two-tailed Student’s t-test; *p* < 0.05 was considered statistically significant. (C) UMAP plot of the developmental trajectory of CD8^+^ T cells. (D) UMAP plots and density plots showing the distribution of CD8^+^ T cells in HD, cEBV^lo^, and cEBV^hi^ groups. (E) UMAP plot depicting the developmental trajectory of NK cells. (F) UMAP plots and density plots displaying the distribution of NK cells in HD, cEBV^lo^, and cEBV^hi^ groups. (G) Boxplots showing the ratio of unswitched memory and switched memory B cells in total PBMCs among HD, cEBV^lo^, and cEBV^hi^ groups. Hinges represent the 25th and 75th percentiles, and whiskers extend to values within 1.5× IQR from the hinges. Horizontal bars indicate the median value. The *p* values were determined by unpaired two-tailed Student’s t-test; *p* < 0.05 was considered statistically significant. (H) Bar plots showing GO enrichment analysis of upregulated biological processes (BP) signaling pathway in unswitched memory and switched memory B cell clusters comparing cEBV^hi^ vs. cEBV^lo^.

To investigate the impact of EBV infection on NKTCL lymphomagenesis, we analyzed EBV gene expression in PBMCs. High EBV gene expression was detected in total PBMCs of three patients, including the latent genes *EBER1* and *LMP1*, early lytic genes *BALF3*, *BALF4*, *BALF5*, and *BILF1*, as well as the late gene *BNRF1* (Figure 4A), indicating a high intracellular EBV load (cEBV^hi^). We also suspected low EBV gene expression in other patients (cEBV^lo^) that could not be detected by scRNA-seq due to its sensitivity. Using the PrimeFlow assay, we confirmed *EBER1/2* and *gp350* expression levels in total PBMCs of cEBV^hi^ and cEBV^lo^ patients (Figure 4B). The EBV genes were mainly expressed in CD8^+^ T cells (GZMK^+^ Tcm, GZMK^+^ GZMB^+^ Tem, GZMK^-^ GZMB^+^ Tem, and KLRC2^+^ Tem), NK cells (CD56^dim^*_BNC2*, CD56^dim^*_DUSP1*, CD56^dim^*_PTPRCAP*, and CD56^dim^*_S100A6*), and NKT-like cells (Figure S7 and S8).

Further analysis revealed that GZMK^+^ GZMB^+^ Tem cells accumulated in the CD8^+^ T cell subset, with a decrease in GZMK^-^ GZMB^+^ Tem cells in cEBV^hi^ patients (Figure 4C and 4D). GZMK^+^ GZMB^+^ Tem cells expressed more cell-killing and cytotoxicity-associated genes compared to GZMK^-^ GZMB^+^ Tem cells (Figure S9A and S9B), and upregulated cell killing and cytotoxicity pathways while downregulating cell differentiation pathways in cEBV^hi^ patients (Figure S9C). According to developmental trajectory analysis, we speculate that the high intracellular EBV load might promote the differentiation of CD8^+^ T cells into GZMK^+^ GZMB^+^ Tem cells with high cytotoxicity, blocking the differentiation into GZMK^-^ GZMB^+^ Tem cells (Figure 4C and 4D).

In the NK cell subset of cEBV^hi^ patients, CD56^dim^*_PTPRCAP* NK cells predominated with high cytotoxicity (*KLRC1*, *GZMH*, and *CX3CR1*), expressing the *LAG3* gene, which is usually used to define exhausted T cells (Figure 4E, 4F, and S10A). These cells upregulated "MHC class II protein complex binding" and "antigen processing and presentation" pathways (Figure S10B and S10C), suggesting an overactivated and exhaustion-like state. B cells, especially classical memory B cells, showed a decrease in PBMCs of cEBV^hi^ patients (Figure 4G and S10D), along with increased B cell activation and differentiation pathways (Figure 4H and S10E).

Overall, PBMCs of cEBV^hi^ patients exhibited high activation and potential cytotoxicity of CD8^+^ T and NK cells, as well as a decrease in memory B cells, indicating a state of immune dysregulation in the blood microenvironment.

Finally, we assessed the cytotoxic activity of NK and CD8^+^ T cells in newly diagnosed NKTCL PBMCs. NK cells were sorted from PBMCs of NKTCL patients (Figure S11A) and co-cultured with CFSE-labeled K562 cells for 4 h. The expression of the cytotoxicity marker CD107a on CFSE^-^ CD56^+^ NK cells was slightly decreased in NKTCL compared to HD (Figure S11B and S11C). The relative death rate of CFSE^+^ K562 cells was higher in the HD group (Figure S11B and S11D), indicating impaired NK cell cytotoxic function in NKTCL patients. Total PBMCs from HD group and NKTCL patients were stimulated with anti-human CD3/CD28 beads to assess CD8^+^ T cell cytotoxicity. CD8^+^ T cells in NKTCL patients showed higher granzyme B expression, which increased significantly after stimulation compared to HD (Figure S11E, S11F, and S11G). IFN-γ^+^ CD8^+^ T cells did not show differences between groups with or without stimulation. These data suggest that CD8^+^ T cells in NKTCL PBMCs exhibit high cytotoxicity levels.

## Discussion

EBV infects over 90% of the global population, with most individuals experiencing mild symptoms or being asymptomatic. However, in some individuals, especially those with weakened immune systems, EBV infection can lead to various cancers, including NKTCL. The reason why EBV only causes malignancies in a small subset of infected individuals has been a longstanding question. Numerous studies have indicated that immune system abnormalities play a role in the development of EBV-associated diseases. Recent multi-omics analyses of NKTCL tumor samples have identified critical genetic and epigenetic alterations, distinct molecular subtypes, and variations in the EBV genome 19-23, 25, 26, offering insights for future therapeutic approaches. However, the comprehensive immune profile of peripheral blood in newly diagnosed NKTCL patients has not been extensively studied. Our current research aims to provide a detailed immunoprofiling atlas of peripheral blood samples from newly diagnosed NKTCL patients. By comparing healthy donors with NKTCL patients, we have identified significant changes in the subsets of proliferative-NK/T cells, classical memory B cells, and GZMK^+^ CD8^+^ Tcm cells, along with increased HLA class II expression in NK cells and enhanced NK-CD4^+^ T cell communication in NKTCL patients' peripheral blood. Notably, patients with high intracellular EBV viral load exhibit more pronounced immune dysregulation than those with low viral load.

The immunoprofiling landscape presented in our current study could provide an informative platform for translational research in NKTCL. We have identified a unique subset of Proli-NK/T cells in the peripheral blood of NKTCL patients, comprising NK cells, CD4^+^ T cells, and CD8^+^ T cells. Unlike the malignant NK cells found in the NKTCL tumor microenvironment 43, these proliferative NK, CD4^+^ T, and CD8^+^ T cells in NKTCL patients do not exhibit genomic instability or chromosomal abnormalities, suggesting a non-malignant phenotype. Gene expression analyses indicate that Proli-NK/T cells are enriched with cell-cycle and IFN-γ-associated genes, reflecting a rapid cell cycle and enhanced IFN-γ response, consistent with the increased production and secretion of IFN-γ observed in NKTCL 44, 45. Cell-to-cell communication analysis did not show exceptional communication between Proli-NK/T cells and other cells, suggesting that specific cytokines in the peripheral blood of NKTCL may trigger the presence of Proli-NK/T cells. Future investigation is required to clarify the function and impact of this unique population on disease progression.

According to the published report, memory B cells are more abundant in healthy donors compared to cancer patients 29. In NKTCL peripheral blood, both unswitched and switched memory B cells were significantly decreased. GO enrichment analysis of scRNA-seq showed that naive, unswitched memory, and switched memory B cells are enriched with death receptor signaling. Flow cytometry confirmed a significant increase in FAS expression on peripheral blood B cells in NKTCL, rendering B cells more prone to apoptosis. Additionally, there was a notable increase in plasmablasts/plasma cells in NKTCL peripheral blood, with elevated expression of the plasma transcription factor PRDM1 detected in switched memory B cells, indicating a tendency for classical memory B cells to differentiate into plasmablasts/plasma cells. Interestingly, three cEBV^hi^ patients showed enrichment of cell population expressing high levels of EBV latent and lytic gene expression, which correlated with a significant reduction in classical memory B cells. Terminal differentiation into plasma cells is tightly associated with EBV replication and lytic reactivation 46-48. Our findings suggest that the predisposition to cell death and differentiation contributes to the decrease in classical memory B cells in NKTCL peripheral blood, with EBV activation playing a potential role in their differentiation.

NK cells reveal little difference in subset proportions between healthy donors and NKTCL patients, but increased activation as indicated by increased expression of activating surface markers NKp46 and NKp30 as well as HLA class II expression in NKTCL NK cells. The MHC class II-derived signaling pathway network indicated enhanced NK cell-CD4^+^ T cell communication in NKTCL. Naive CD4^+^ T cells mainly polarize towards Th1 in NKTCL. Notably, a GZMK^+^ CD8^+^ Tcm cell subpopulation increased in NKTCL compared to healthy donors. Recent studies suggest that GZMK^+^ CD8^+^ T cells exhibit a vital role in inflammation associated with various diseases 49-51. Upregulation of granzyme K in CD8^+^ T cells has also been reported in EBV-associated gastric cancer and is implicated in EBV infection 52. This unique cell subset in NKTCL is enriched with signaling related to IFN-γ responses, suggesting that the emergence of GZMK^+^ CD8^+^ Tcm cells may contribute to the immune microenvironment of NKTCL peripheral blood. Further analysis is needed to identify the pathogenic role of GZMK^+^ CD8^+^ T cells in NKTCL.

Based on the transcriptional profile of EBV genes detected by scRNA-seq, we stratified NKTCL patients into cEBV^hi^ and cEBV^lo^. Comparative analysis revealed that three cEBV^hi^ patients exhibited an immune dysregulation state. CD8^+^ T cells displayed a cell differentiation arrest at the GZMK^+^ GZMB^+^ Tem stage, which showed enhanced cell killing and cytotoxicity, while NK cells tended to differentiate into the CD56^dim^*_PTPRCAP* stage, expressing a high level of cytotoxicity markers and* LAG3* and possessing stronger antigen processing and presentation capability, indicative of exhaustion-like state and dysfunction. Exhaustion and functional defects of NK cells are linked to poor survival in newly diagnosed multiple myeloma patients 53, and a low peripheral blood NK cell count is associated with impaired survival in patients with follicular lymphoma 54. These suggest that immune dysregulation in the peripheral blood of NKTCL might be correlated with EBV-positive cell populations with active viral replication.

One limitation of our study is the small number of cEBV^hi^ individuals in the cohort. EBV lytic reactivation can impact cellular signaling pathways and alter cellular responses in peripheral blood. Analyzing a larger number of cEBV^hi^ patients could strengthen our conclusions. The overall cohort size was modest, with 12 healthy donors and 20 newly diagnosed NKTCL patients. Future studies should include longitudinal samples to monitor immune changes during treatment.

## Conclusion

We collected PBMCs from 12 healthy donors and 20 newly diagnosed NKTCL patients to investigate changes in PBMC profiling among NKTCL patients. Our study provides insights into immune alterations in NKTCL patients’ peripheral blood, highlighting that Proli-NK/T and GZMK^+^ CD8^+^ Tcm cells increase, classical memory B cells decrease, upregulation of HLA class II in NK cells enhances NK-CD4^+^ T cell communication, and intracellular EBV viral load might be related to immune dysregulation in NKTCL. Further research is warranted to understand the implications of these immune changes in NKTCL pathogenesis and potential immune-based therapies.

## Supplementary Material

Supplementary figures.

Supplementary table 1.

Supplementary table 2.

Supplementary table 3.

## Abbreviations

NKTCL: natural killer/T-cell lymphoma
EBV: Epstein-Barr virus
EBERs: EBV-encoded small RNAs
EBNA1: EBV nuclear antigen 1
LMPs: latent membrane proteins
R/R: relapsed/refractory
scRNA-seq: single-cell RNA sequencing
DEGs: differential expression genes
CNVs: copy number variations
PBMCs: peripheral blood mononuclear cells
cDC: conventional dendritic cells
pDC: plasmacytoid dendritic cells
NK: natural killer
MAIT: mucosal-associated invariant T
Tcm: central memory T cell
Tem: effector memory T cell
GZMK/GZMB/GZMH: granzyme K/B/H
IFN-γ: interferon-gamma
