# Supplementary material for: Peripheral blood immune profiling reveals key signatures in newly diagnosed NK/T cell lymphoma patients
Source: Theranostics. 2026 Jun 17;16(13):7626–40. doi: 10.7150/thno.132582 (PMC13295915; doi:10.7150/thno.132582)
Supplement: Supplementary file 1 — Supplementary figures. [file thnov16p7626s1.pdf]

Supplemental Figures

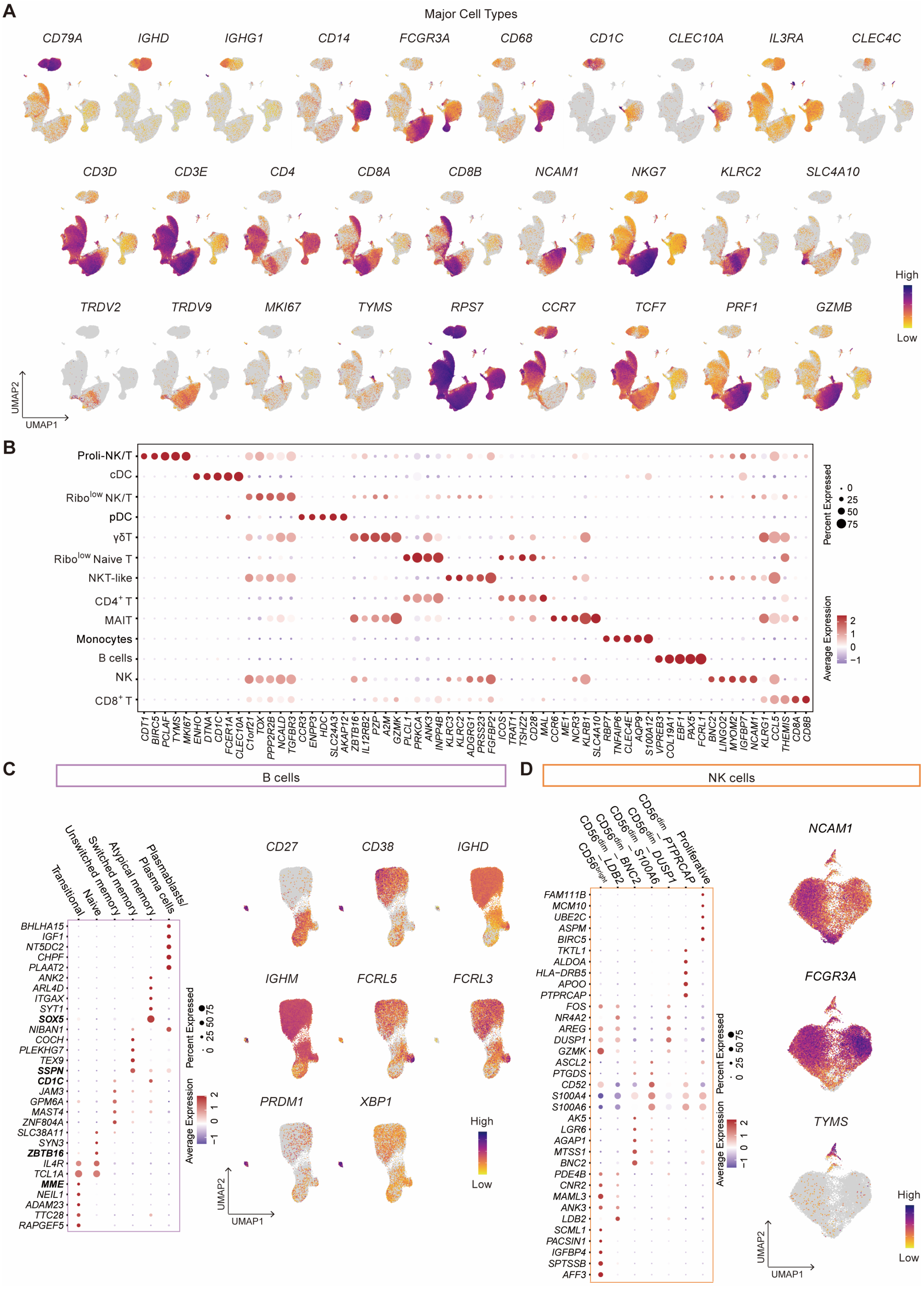

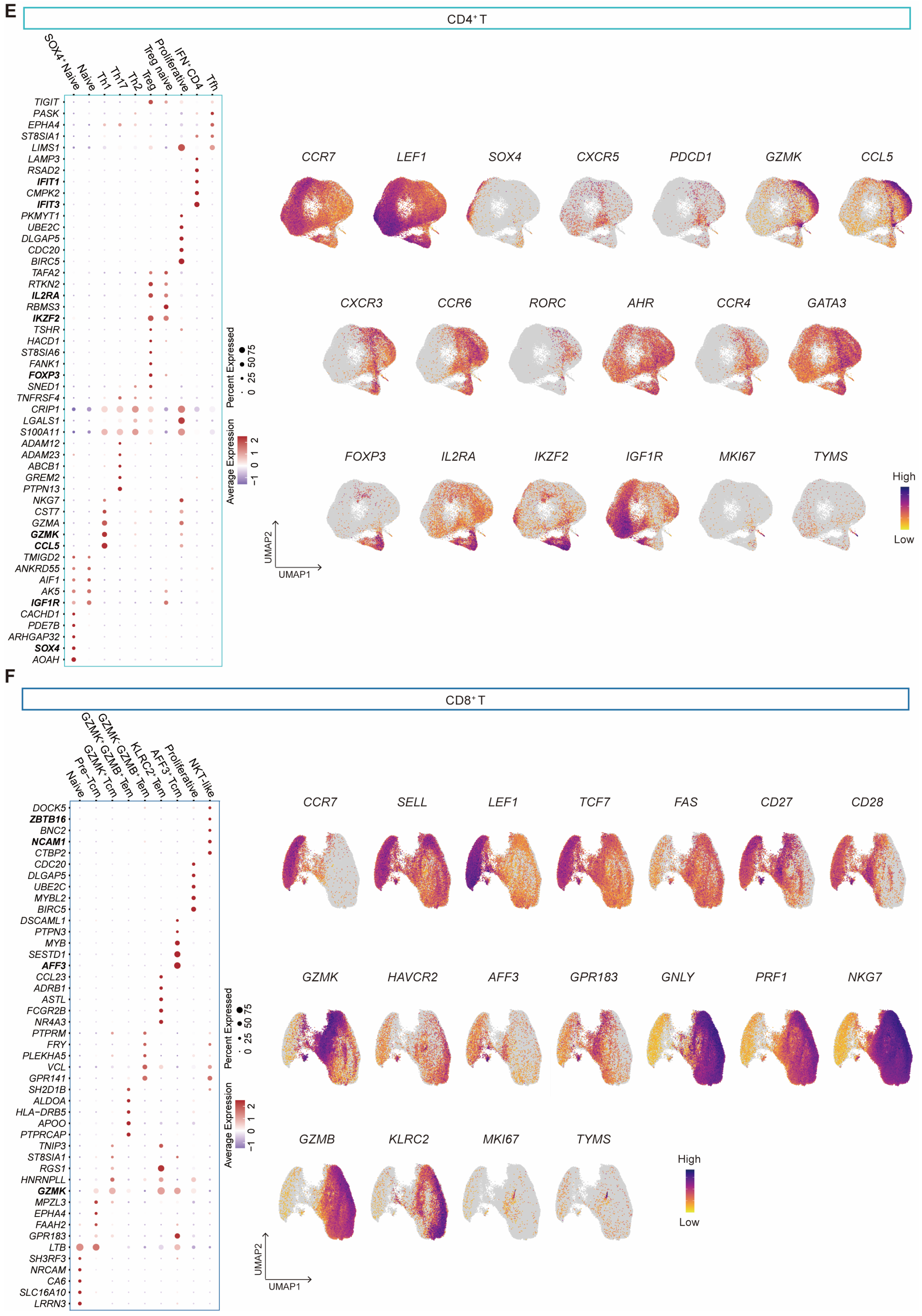

Figure S1. Cell markers of major cell types and 32 distinct subsets (related to Figure 1)

(A) UMAP plots showing the distribution of expression of selected signature genes for

major cell types.

(B) Bubble plots showing the expression of marker genes for major cell types.

(C-F) Bubble plots and UMAP plots displaying the expression of selected marker genes for B cell, NK cell, $CD4^+$ T cell, and $CD8^+$ T cell subsets.

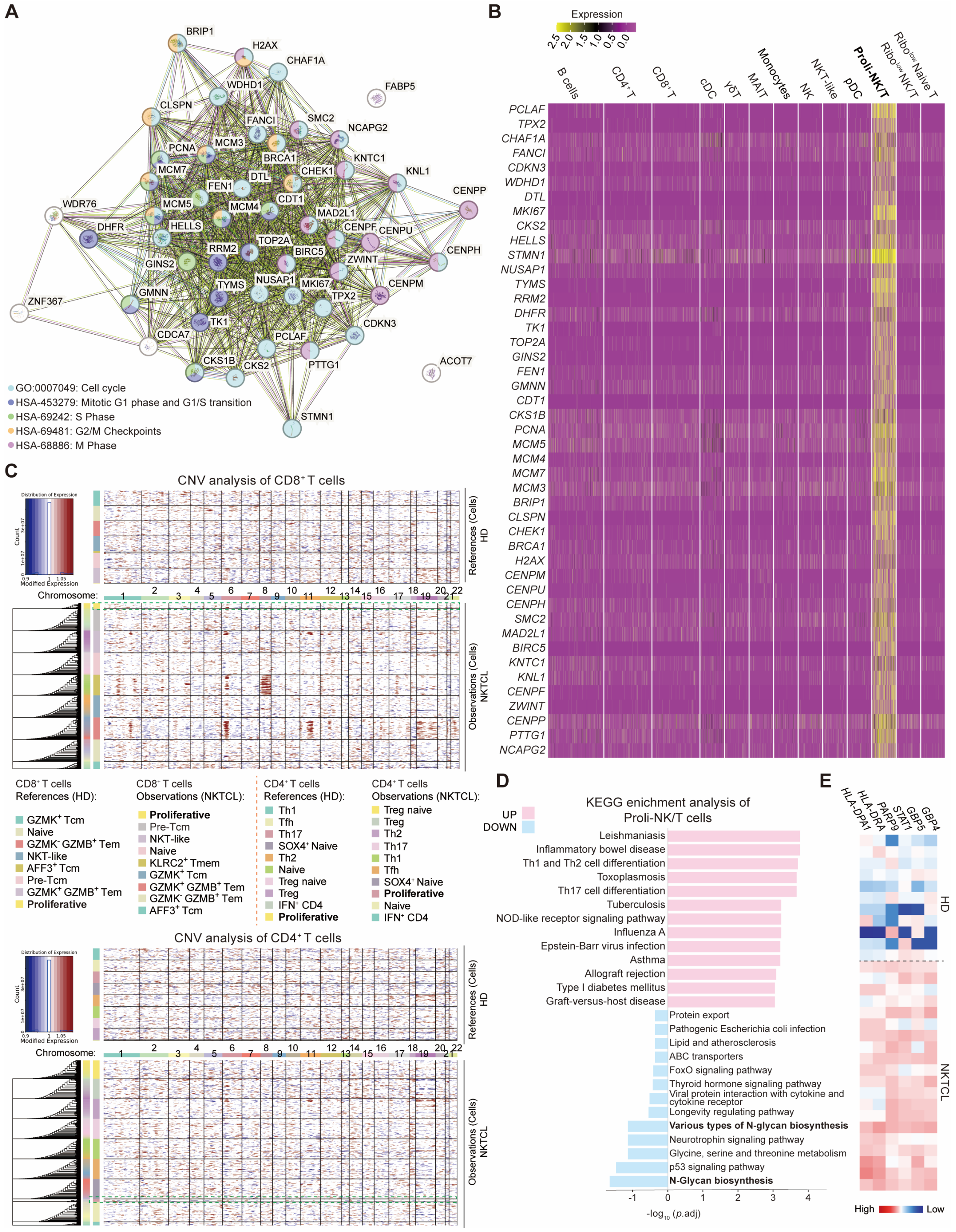

Figure S2. Characteristic gene expression of Proli-NK/T cells and CNV analysis (related to Figure 1)

(A) Top 50 gene interaction network of Proli-NK/T cells using the STRING database.

(B) Heatmap of cell cycle-associated gene expression in major cell types.

(C) Heatmap showing the CNV analysis of CD8^{+} T and CD4^{+} T cell subsets. Red and

blue colors represent high and low CNV levels, respectively.

(D) Bar plots showing the KEGG enrichment analysis comparing the changes of NKTCL vs. HD in Proli-NK/T cells.

(E) Heatmap showing the 6 upregulated gene expression in Proli-NK/T cells.

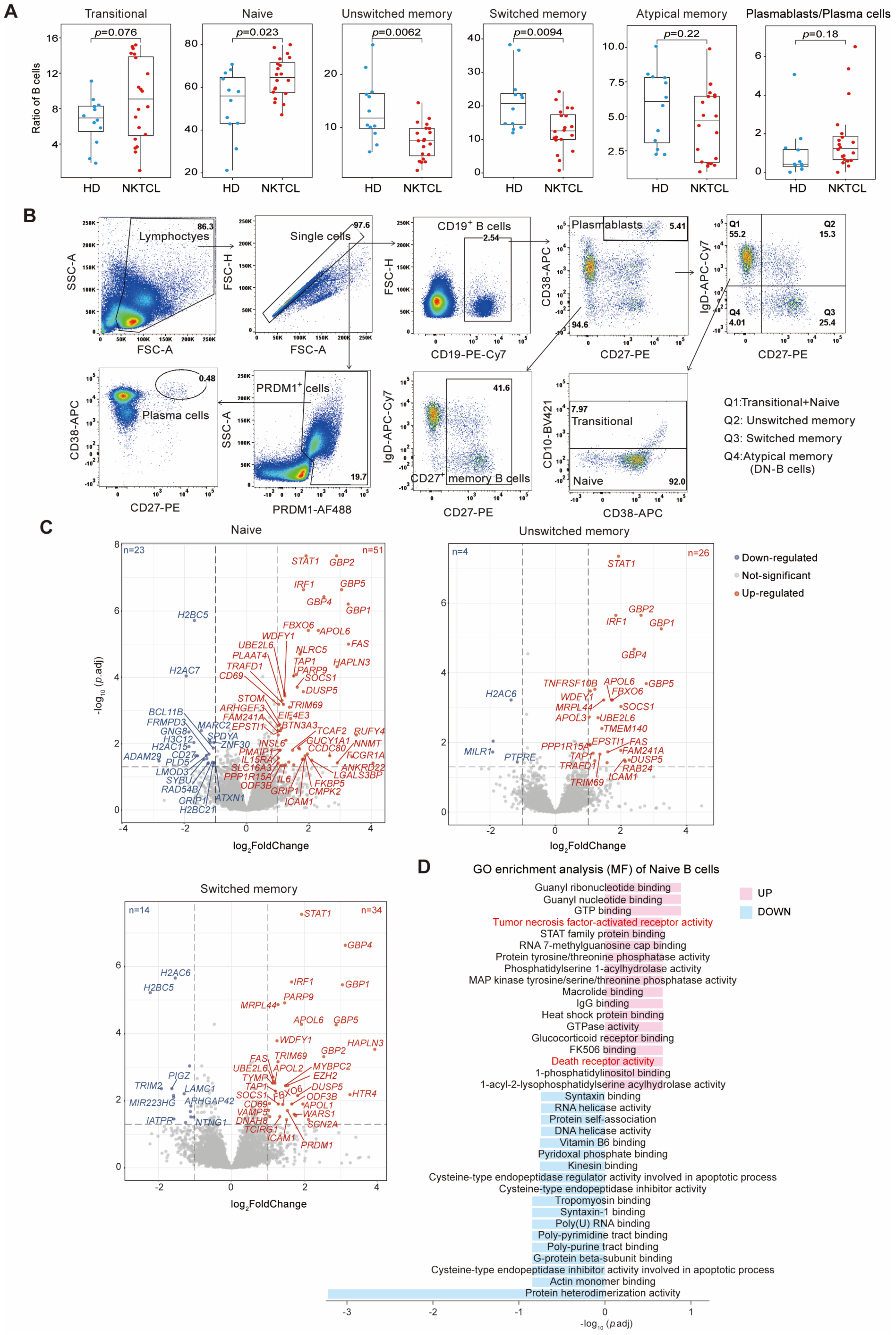

Figure S3. B cell subset characteristics and flow cytometry gating strategy (related to Figure 2)

(A) Boxplots showing the ratio of each B cell subsets in HD and NKTCL groups. Hinges represent the 25th and 75th percentiles, and whiskers extend to values within 1.5× IQR from the hinges. Horizontal bars indicate the median value. The *p*-value were determined using an unpaired two-tailed Student's t-test, $p < 0.05$ was considered statistically significant.

(B) Flow cytometry gating strategy for B cell subsets and PRDM1^{+} CD27^{+} memory B cells.

(C) Volcano plots showing the significantly changed genes (p.adj < 0.05) in naive, unswitched memory, and switched memory B cell clusters.

(D) Bar plots presenting the GO enrichment analysis of molecular function (MF) signaling pathways comparing the changes in NKTCL vs. HD in the naive B cell cluster.

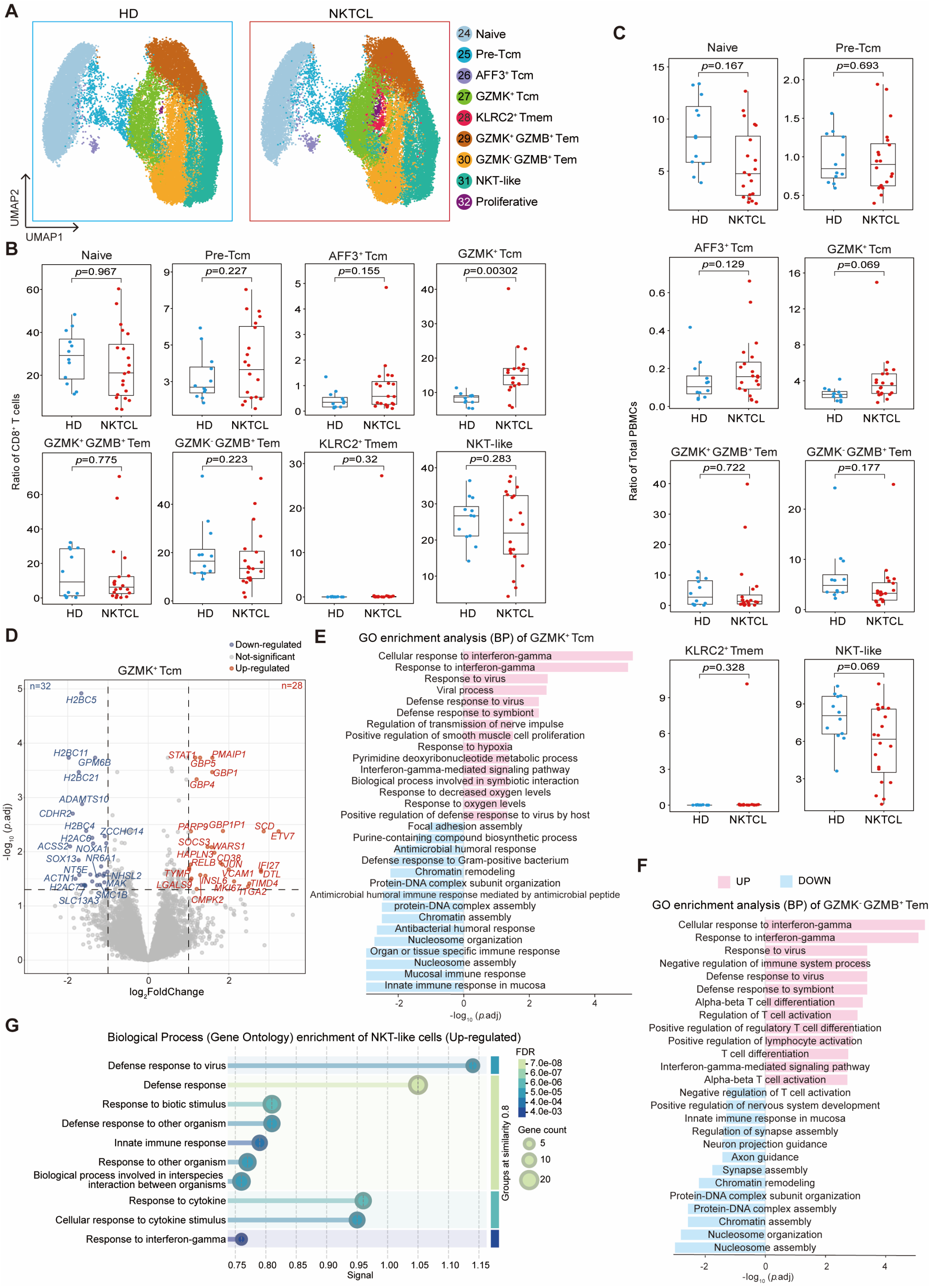

Figure S4. Differential CD8^{+} T cell composition between NKTCL and HD groups

(A) UMAP plots displaying the variation in CD8^{+} T cell subsets between HD and NKTCL groups.

(B) Boxplots depicting the proportion of each subset in CD8^{+} T cells in HD and NKTCL

groups.

(C) Boxplots showing the ratio of each $CD8^+$ T cell subset in total PBMCs in HD and NKTCL groups.

(D) Volcano plots showing the significantly changed genes (p.adj < 0.05) in $GZMK^+$ Tcm cells.

(E-F) Bar plots presenting GO enrichment analysis of biological process (BP) signaling pathways comparing changes in $GZMK^+$ Tcm and $GZMK^-$ $GZMB^+$ Tem cells between NKTCL and HD groups.

(G) Bar plots displaying GO enrichment analysis of upregulated biological process (BP) signaling pathways comparing the changes in NKT-like cells between NKTCL and HD groups using the STRING database.

In (B) and (C), hinges represent the 25th and 75th percentiles, and whiskers extend to values within 1.5× IQR from the hinges. Horizontal bars indicate the median value. The p-value were determined using a general linear model with the effect of age involved; $p < 0.05$ was considered statistically significant.

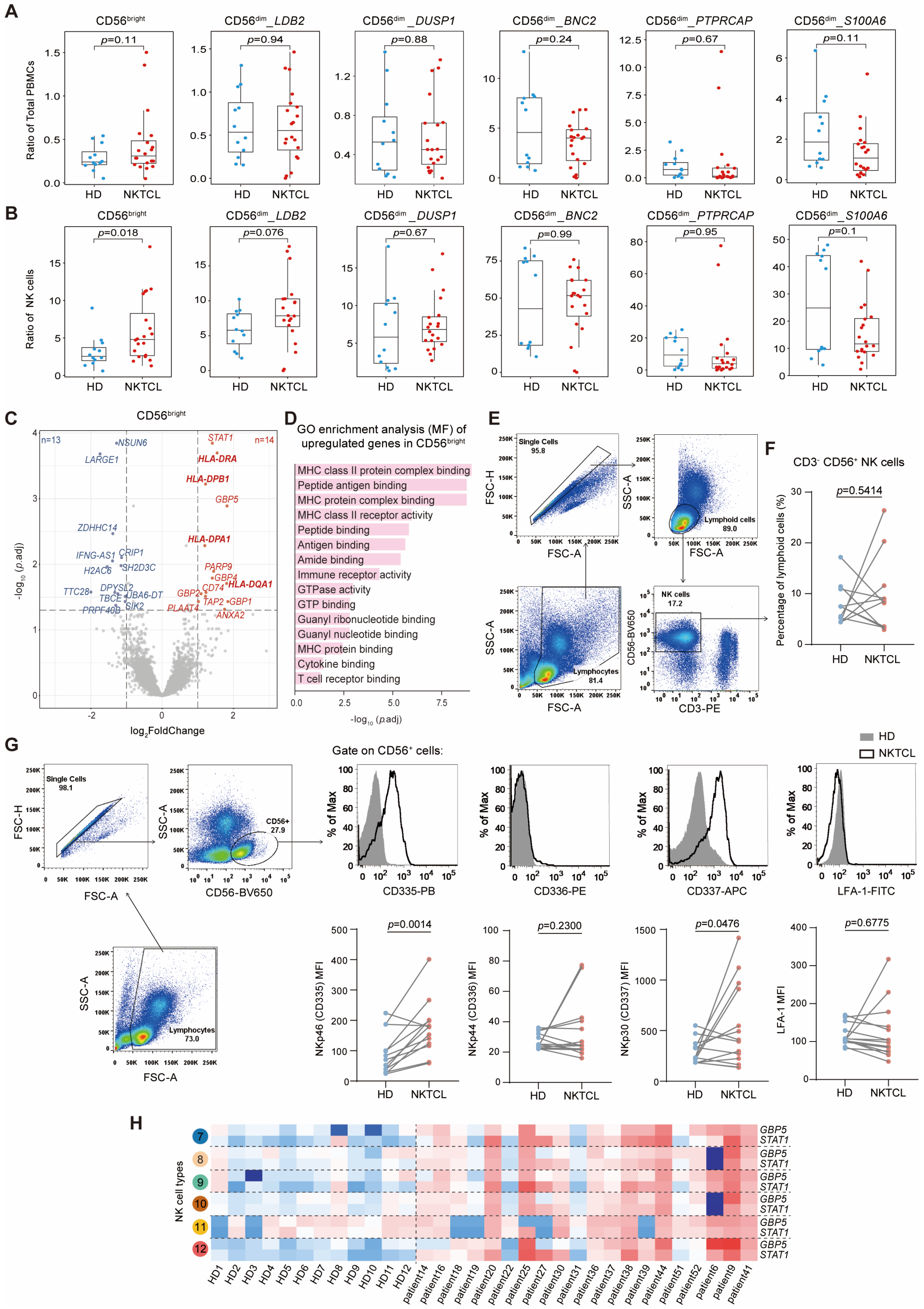

Figure S5. NK cell subsets and flow cytometry analysis (related to Figure 3)

(A) Boxplots showing the ratio of each NK cell subset in total PBMCs for HD and NKTCL groups.

(B) Boxplots showing the ratio of each subset in NK cells for HD and NKTCL groups.

(C) Volcano plots showing the significantly changed genes (p.adj < 0.05) in the CD56^{bright} cluster.

(D) Bar plots showing the GO enrichment analysis of upregulated molecular function (MF) signaling pathways to compare changes in NKTCL vs. HD in the CD56^{bright} cluster.

(E) Flow cytometry gating strategy for NK cells.

(F) Flow cytometry analysis showing the frequency of NK cells among lymphoid cells in HD (n=7) and NKTCL (n=11) groups.

(G) FACS analysis showing the activation markers of NK cells in HD (n=12) and NKTCL (n=14) groups.

(H) Heatmap displaying the expression of *GBP5* and *STAT1* in NK cell subsets.

In (A) and (B), hinges represent the 25th and 75th percentiles, and whiskers extend to values within 1.5× IQR from the hinges. Horizontal bars indicate the median value. The p-values were determined by unpaired two-tailed Student's t-test. In (F) and (G), the p-values were determined by paired two-tailed Student's t-test. $p < 0.05$ was considered statistically significant.

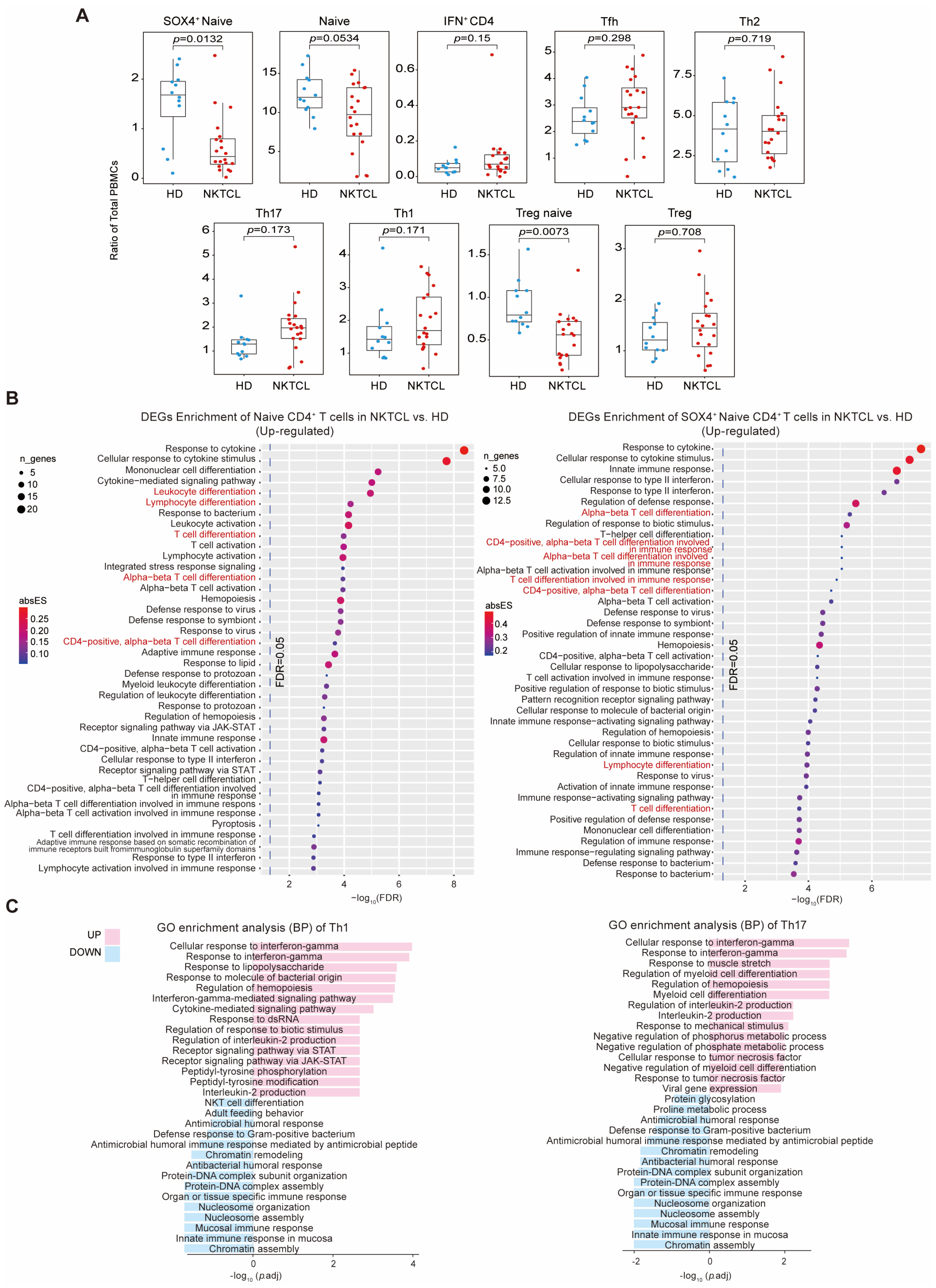

Figure S6. Statistic and signaling pathway enrichment of CD4$^+$ T cell subsets (related to Figure 3)

(A) Boxplots showing the ratio of each CD4$^+$ T cell subset in total PBMCs in HD and NKTCL groups. Hinges represent the 25th and 75th percentiles, and whiskers extend

to values within 1.5× IQR from the hinges. Horizontal bars indicate the median value. The *p*-values were determined using a general linear model with the effect of age included; $p < 0.05$ was considered statistically significant.

(B) Bubble plots showing the GO enrichment analysis of upregulated biological process (BP) signaling pathways for comparing the changes in NKTCL vs. HD in naive and SOX4^{+} naive CD4^{+} T cells.

(C) Bar plots showing the GO enrichment analysis of biological process (BP) signaling pathways for comparing the changes in NKTCL vs. HD in Th1 and Th17 cells.

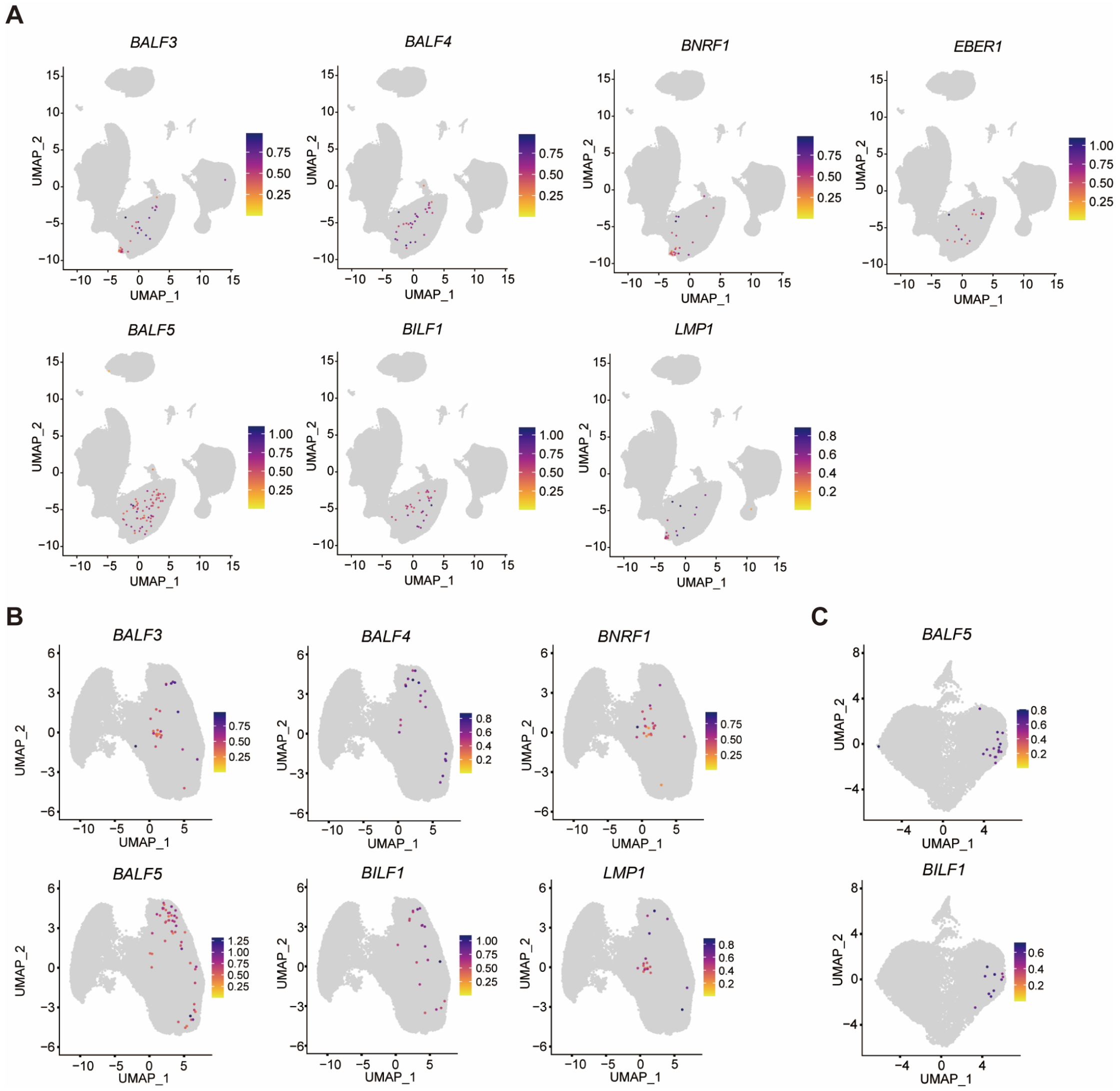

Figure S7. UMAP plots showing the distribution of EBV gene expression in major cell types (A), CD8^{+} T cell subsets (B), and NK cell subsets (C) (related to Figure 4)

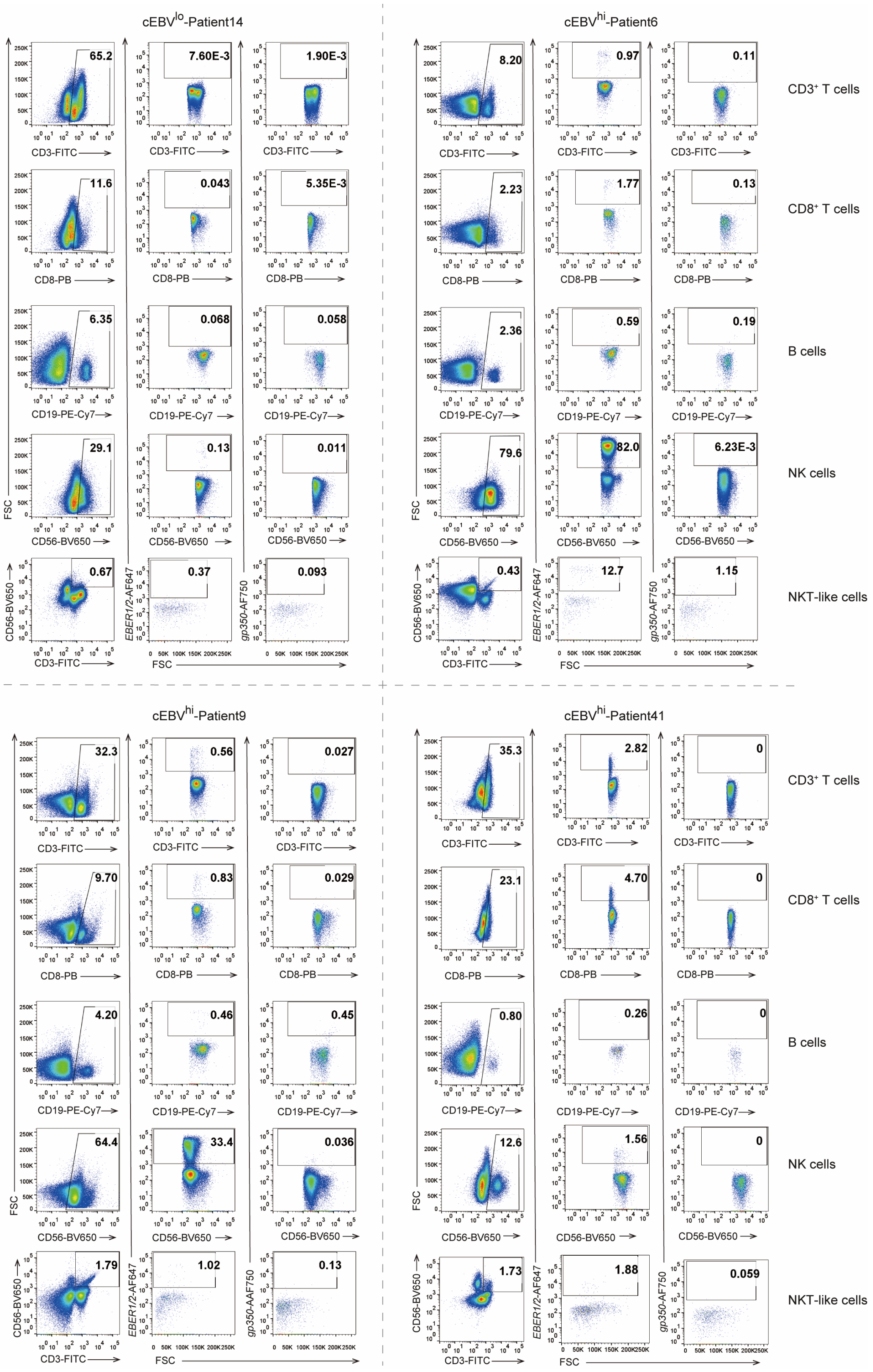

Figure S8. PrimeFlow assay showing the expression of *EBER1/2* and *gp350* in CD3^{+} T, CD8^{+} T, B, NK, and NKT-like cells of cEBV^{lo} and cEBV^{hi} NKTCL patients (related to Figure 4)

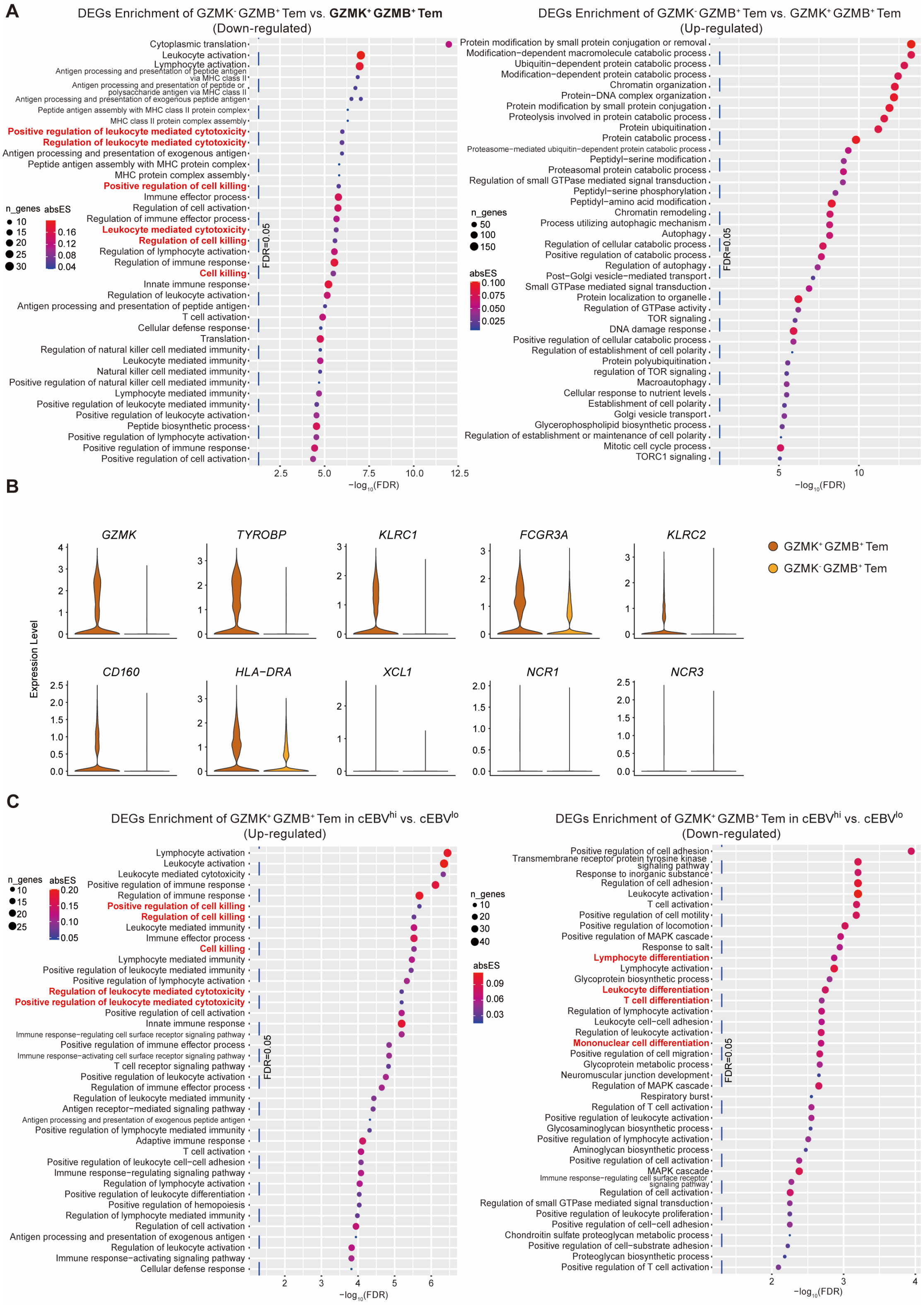

Figure S9. Characteristics of GZMK^{+} GZMB^{+} Tem cells (related to Figure 4)

(A) Bubble plots showing the different signaling pathway enrichment of characteristic genes between GZMK^{+} GZMB^{+} Tem and GZMK^{-} GZMB^{+} Tem cells.

(B) Violin plots showing the expression of cell-killing associated genes in $GZMK^+$ $GZMB^+$ Tem and $GZMK^-$ $GZMB^+$ Tem cells.

(C) Bubble plots showing the GO enrichment analysis of biological process (BP) signaling pathways for comparing $cEBV^{hi}$ vs. $cEBV^{lo}$ in $GZMK^+$ $GZMB^+$ Tem cells.

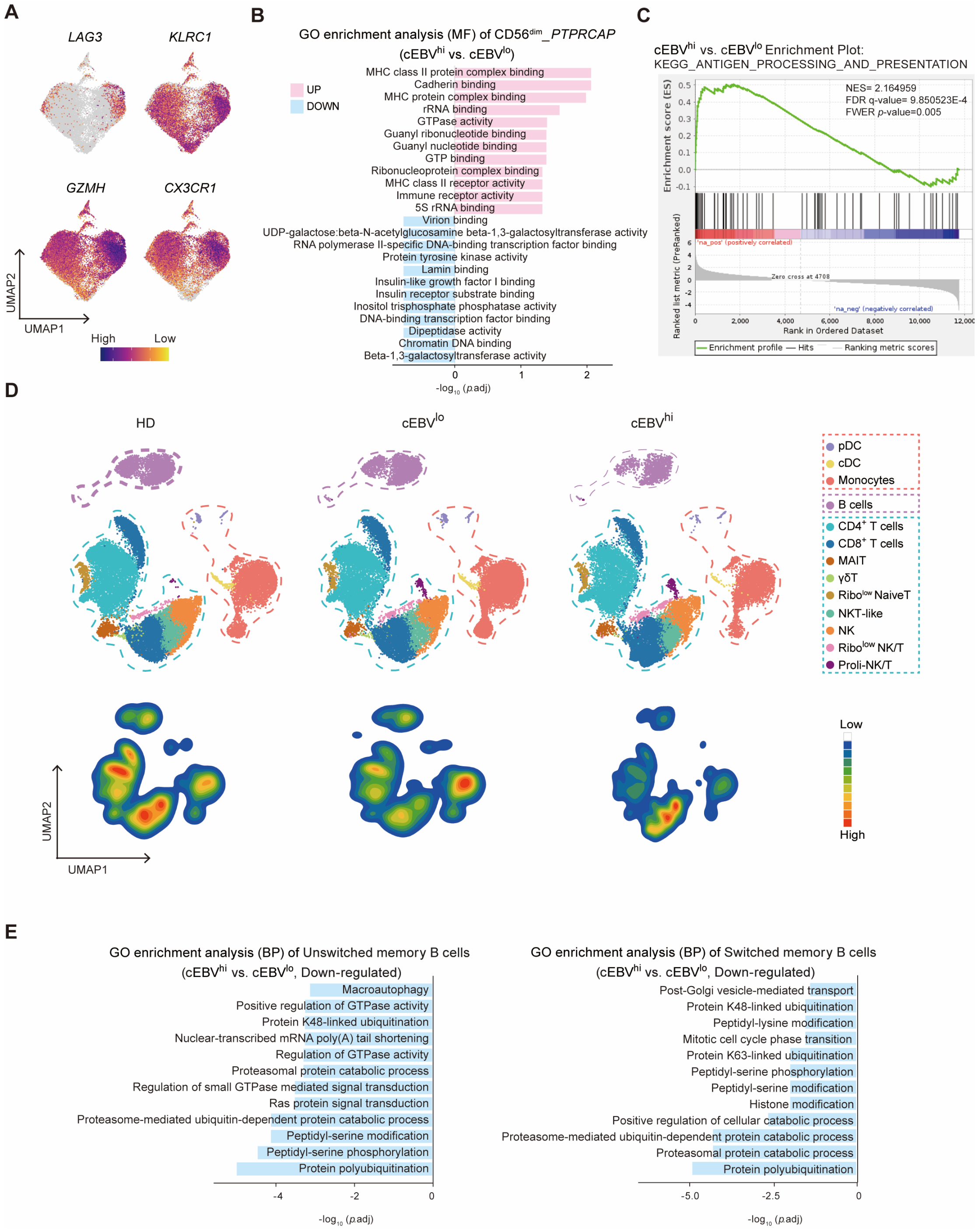

Figure S10. Characteristics of CD56^{dim}_*PTPRCAP* NK cells and classical memory B cells in cEBV^{hi} NKTCL patients (related to Figure 4)

(A) UMAP plot showing the distribution of *LAG3*, *KLRC1*, *GZMH*, and *CX3CR1* gene expression in NK cell subsets.

(B) Bar plots depicting the GO enrichment analysis of molecular function (MF) signaling pathways to compare the changes in cEBV^{hi} vs. cEBV^{lo} in

CD56^{dim}_*PTPRCAP* NK cells.

(C) Gene set enrichment analysis (GSEA) plot for the antigen processing and presentation pathway in cEBV^{hi} NKTCL patients of CD56^{dim}_*PTPRCAP* NK cells.

(D) UMAP plots and UMAP density plots characterizing the distribution of major cell types among HD, cEBV^{lo}, and cEBV^{hi} groups.

(E) Bar plots showing the GO enrichment analysis of downregulated biological processes (BP) signaling pathways to compare the changes in cEBV^{hi} vs. cEBV^{lo} in unswitched memory and switched memory B cell clusters.

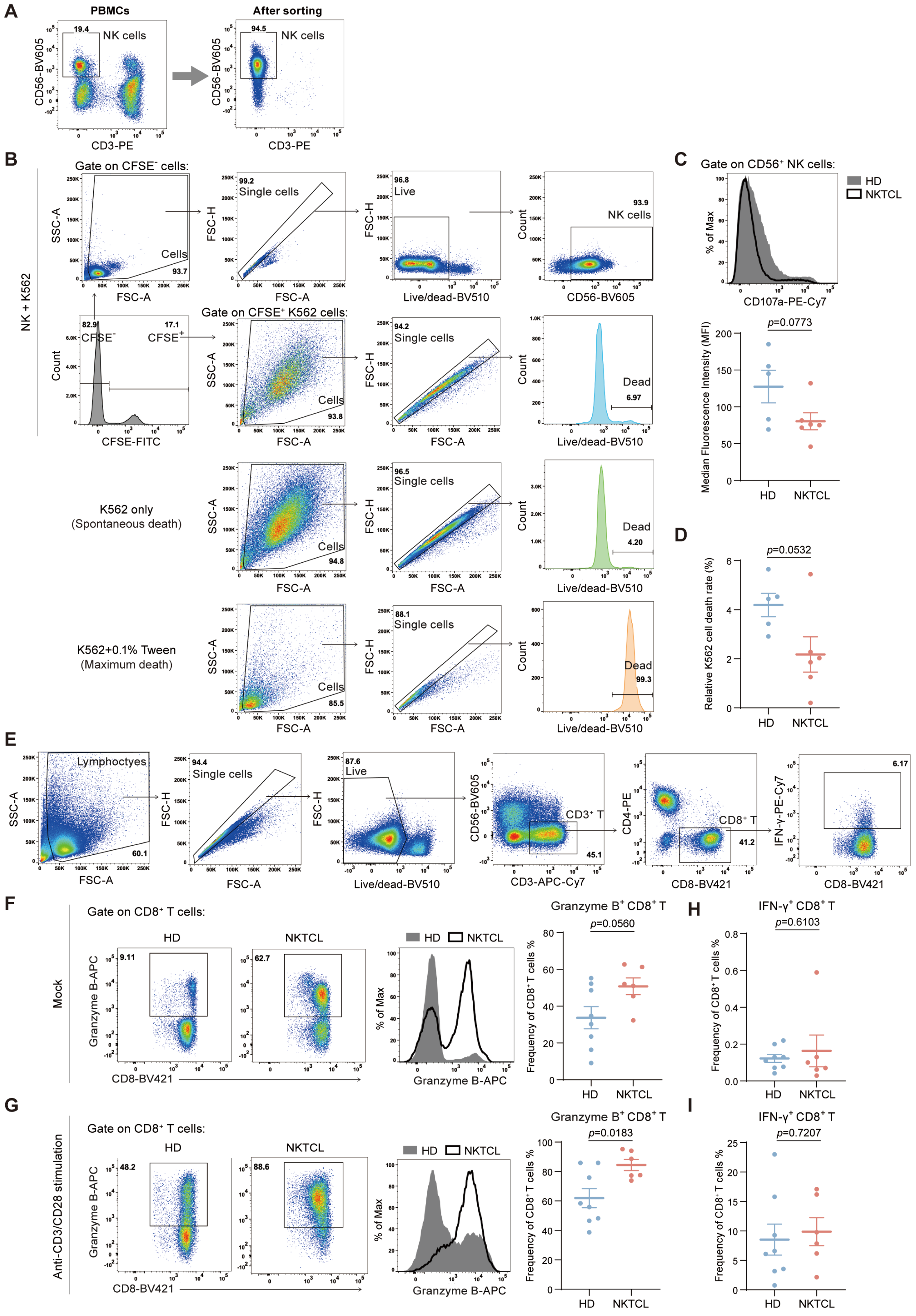

Figure S11. Functional validation assays of NK and CD8$^+$ T cells from NKTCL PBMCs

(A) Flow cytometry plots showing the CD3$^-$ CD56$^+$ NK cells before and after sorting

from PBMCs.

(B) Flow cytometry gating strategy for NK cell cytotoxicity assay. Sorted NK cells were co-cultured with CFSE-labeled K562 cells at a 5:1 effector:target cell (E:T) ratio for 4 h. NK cell cytotoxicity was assessed by detecting the expression level of CD107a after gating on $CFSE^-$ NK cells and measuring the death rate of $CFSE^+$ K562 cells.

(C) FACS analysis showing the expression of CD107a in $CFSE^-$ NK cells in HD (n=5) and NKTCL (n=6) groups.

(D) Flow cytometry analysis showing relative the death rate of $CFSE^+$ K562 cells in HD (n=5) and NKTCL (n=6) groups. Relative cell death rate (%) = [(experimental death rate-spontaneous death rate) / (maximum death rate-spontaneous death rate)] × 100.

(E) Flow cytometry gating strategy for $CD8^+$ T cells from total PBMCs after anti-human CD3/CD28 beads stimulation for 24 h.

(F and G) Flow cytometry analysis of the percentage of granzyme B^+ cells in $CD8^+$ T cells in HD (n=8) and NKTCL (n=6) groups without (F) or with (G) anti-human CD3/CD28 beads stimulation.

(H and I) Flow cytometry analysis of the percentage of IFN-γ^+ cells in $CD8^+$ T cells in HD (n=8) and NKTCL (n=6) groups without (H) or with (I) anti-human CD3/CD28 beads stimulation.

In (C), (D), (F), (G), (H) and (I), data were presented as mean ± s.e.m; the *p*-values were determined by unpaired two-tailed Student's t-test, and $p < 0.05$ was considered statistically significant.
