# Supplementary material for: Peripheral blood immune profiling reveals key signatures in newly diagnosed NK/T cell lymphoma patients
Source: Theranostics. 2026 Jun 17;16(13):7626–40. doi: 10.7150/thno.132582 (PMC13295915; doi:10.7150/thno.132582)
Supplement: Supplementary file 4 — Supplementary table 3. [file thnov16p7626s4.pdf]

KEY RESOURCE TABLE

REAGENT or RESOURCE	SOURCE	IDENTIFIER
**Antibodies**		
Fc Block	BD Biosciences	Cat# 564220; RRID: AB_2728082
CD107a-PE-Cy7	BD Biosciences	Cat# 561348; RRID: AB_10644018
CD3-FITC	Biolegend	Cat# 317305; RRID: AB_571906
CD3-PE	Biolegend	Cat# 300408; RRID: AB_314062
CD3-APC-Cy7	Biolegend	Cat# 300426; RRID: AB_830755
CD4-PE	Biolegend	Cat# 300507; RRID: AB_314075
CD8-PB	Biolegend	Cat# 344717; RRID: AB_10551616
CD8-BV421	Biolegend	Cat# 344747; RRID: AB_2629583
CD10-BV421	Biolegend	Cat# 312218; RRID: AB_2561833
CD11a/CD18 (LFA-1)-FITC	Biolegend	Cat# 363416; RRID: AB_2728359
CD27-PE	Biolegend	Cat# 356406; RRID: AB_2561825
CD335 (NKp46)-PB	Biolegend	Cat# 331912; RRID: AB_2149280
CD336 (NKp44)-PE	Biolegend	Cat# 325107; RRID: AB_756099
CD337 (NKp30)-APC	Biolegend	Cat# 325209; RRID: AB_2149450
CD38-APC	Biolegend	Cat# 356606; RRID: AB_2561902
CD56-BV650	Biolegend	Cat# 318344; RRID: AB_2563838
CD56-BV605	Biolegend	Cat# 318334; RRID: AB_2561912
CD95-FITC	Biolegend	Cat# 305605; RRID: AB_314543
Granzyme B-APC	Biolegend	Cat# 372203; RRID: AB_2687027
HLA-DR, DP, DQ-FITC	Biolegend	Cat# 361705; RRID: AB_2563191
IgD-APC-Cy7	Biolegend	Cat# 348218; RRID: AB_11203722
IFN-γ-PE-Cy7	Biolegend	Cat# 502527; RRID: AB_1626154
CD19-PE-Cy7	eBioscience	Cat# 25-0199-42; RRID: AB_1582278
BLIMP1/PRDM1-AF488	Novus	Cat# NB600-235AF488; RRID: AB_3193801
**Chemicals, peptides, and recombinant proteins**		
Ficoll-Paque PLUS	Cytiva	Cat# 17144002
Human Herpes Virus 4 *EBER1/2* target probe	Invitrogen	Cat# PF-210 (VF1-12409)
Human Herpes Virus 4 *gp350* target probe	Invitrogen	Cat# PF-210 (VP47VWR)
10× PBS	Cellmax	Cat# CBS103.05
Bovine Serum Albumin (BSA)	Yeasen Biotech	Cat# 36101ES76
Fetal Bovine Serum (FBS)	Gibico	Cat# A5670701
**Critical commercial assays**		

BD Cytofix/Cytoperm™ Plus Fixation/Permeabilization Solution Kit with BD GolgiStop™	BD Pharmingen	Cat# 554715
PrimeFlow™ RNA Assay Kit	Invitrogen	Cat# 88-18005-210
Human NK Cell Isolation Kit	Miltenyi Biotec	Cat# 130-092-657
LIVE/DEAD™ Fixable Aqua Dead Cell Stain Kit	Invitrogen	Cat# L34966
Dynabeads™ Human T-Activator CD3/CD28	Gibco	Cat# 11161D
**Deposited data**		
Raw scRNA-seq data of human PBMCs	This paper	Sequence Read Archive (SRA): PRJNA1370270
Processed scRNA-seq data of human PBMCs	This paper	NCBI Gene Expression Omnibus (GEO): GSE318371
**Software**		
GraphPad Prism 10	GraphPad Software Inc.	https://www.graphpad.com/
FlowJo	Tree Star Inc.	https://www.flowjo.com/
Adobe Illustrator	Adobe Inc.	https://www.adobe.com/
BioRender	BioRender	https://www.biorender.com/
**Other**		
Celesta flow cytometer	BD Biosciences	N/A
